# Identification of *DELLA* and *GID1* genes in *Catharanthus roseus* and their potential role in regulating vindoline biosynthesis

**DOI:** 10.1007/s11103-025-01599-1

**Published:** 2025-06-05

**Authors:** Lauren F. Cole-Osborn, Natalie Soens, Diana Bernal-Franco, Olga Prifti, Erin J. Cram, Carolyn W. T. Lee-Parsons

**Affiliations:** 1 Department of Chemical Engineering, Northeastern University, Boston, MA 02115 USA; 2 Department of Bioengineering, Northeastern University, Boston, USA; 3 Department of Biology, Northeastern University, Boston, USA; 4 Department of Chemistry and Chemical Biology, Northeastern University, Boston, USA

**Keywords:** DELLA, GID1, *Catharanthus roseus*, Vindoline, Terpenoid indole alkaloid, Paclobutrazol

## Abstract

DELLAs are key regulators of plant growth and development, negatively regulating gibberellic acid (GA) signaling and positively regulating light and jasmonate signaling and juvenile leaf development. In the presence of GA, GID1s bind to DELLAs and signal for their degradation. Here, we identified and characterized *DELLA* and *GID1* genes in *Catharanthus roseus*, the natural source of chemotherapy drugs vinblastine and vincristine. We hypothesized that CrDELLAs positively regulate vindoline biosynthesis, a precursor of vinblastine and vincristine, accumulating in light- and jasmonate-exposed young leaves. To explore this hypothesis, we silenced *CrDELLA* or *CrGID1* genes using virus-induced gene silencing. *CrDELLA*-silenced plants were elongated while *CrGID1*-silenced plants were dwarfed, consistent with their roles in GA-mediated growth. In the first experiment, *CrDELLA*-silencing significantly decreased vindoline pathway gene expression while *CrGID1*-silencing significantly increased vindoline, catharanthine, ajmalicine, and serpentine accumulation. However, subsequent experiments found little to no effect. *C. roseus* seedlings treated with paclobutrazol, an inhibitor of GA biosynthesis shown to increase DELLA protein stability, also provided some evidence for CrDELLA’s positive role in regulating vindoline pathway gene expression. Finally, overexpressed stabilized, N-terminal truncated CrDELLAs in *C. roseus* seedlings yielded significant increases in vindoline pathway promoter activity (*NMT*, *D4H*). Overall, these experiments provide weak to moderate evidence for CrDELLAs positively regulating vindoline biosynthesis. Future experiments with transgenic approaches could strengthen the evidence and clarify this relationship. Activation of the vindoline pathway with stabilized CrDELLAs could increase the production of critical chemotherapeutics, vinblastine and vincristine.

**Supplementary Information:**

The online version contains supplementary material available at 10.1007/s11103-025-01599-1.

## Key message

Transient silencing of *CrDELLA*s and *CrGID1*s, overexpression of truncated *CrDELLA*s, and application of the gibberellic acid-inhibitor paclobutrazol provide weak to moderate evidence that CrDELLAs activate vindoline biosynthesis in *Catharanthus roseus*.

**Supplementary Information:**

The online version contains supplementary material available at 10.1007/s11103-025-01599-1.

## Introduction

*Catharanthus roseus* is the natural source of the valuable chemotherapy medicines, vinblastine and vincristine (Noble 1990). The terpenoid indole alkaloid (TIA) biosynthetic pathway leading to vinblastine and vincristine is complex, requiring over 30 enzymes, transport within cellular compartments and among multiple cell-types, and competing flux towards variable end-products (reviewed in (Kulagina et al. 2022). Overexpression of transcription factors that activate multiple steps in TIA biosynthesis could increase flux towards vinblastine and vincristine, increasing supply of these critical medicines. The upstream TIA biosynthetic pathway is directly regulated by jasmonic acid (JA) signaling transcription factors: MYC2, OCTADECANOID-RESPONSIVE CATHARANTHUS AP2-DOMAIN (ORCAs), and BHLH IRIDOID SYNTHESIS (BISs). However, these transcription factors do not directly regulate the downstream vindoline pathway (consisting of seven enzymes: T16H2, 16OMT, T3O, T3R, NMT, D4H, and DAT) (Colinas et al. 2021; Menke et al. 1999; Paul et al. 2017; Schweizer et al. 2018; Singh et al. 2020, 2021; van der Fits and Memelink 2000a; Van Moerkercke et al. 2015; Moerkercke et al. 2016). Identifying and engineering the transcription factors that regulate the vindoline pathway could overcome this bottleneck and increase production of vinblastine and vincristine.

The vindoline pathway is known to be regulated by light, JA, developmental stage, and their interactions. In regulation by light, the vindoline pathway is repressed by the PHYTOCHROME INTERACTING FACTOR CrPIF1 in the dark and activated by CrGATA1 in the light (Liu et al. 2019). In addition, the vindoline pathway is inducible by JA (Aerts et al. 1994; Besseau et al. 2013; Góngora-Castillo et al. 2012; Hernández-Domínguez et al. 2004; Liscombe et al. 2010a; Raina et al. 2012; van der Fits & Memelink, 2000b; F. A. Vázquez-Flota and De Luca 1998; Wang et al. 2010; Wei 2010; Zhou et al. 2015), but this inducibility is highly dependent on light and developmental state. For example, *D4H* transcript levels in seedlings were induced by JA only in the presence of light, whereas the expression of the upstream enzyme *TDC* was activated by JA even in the dark (F. A. Vázquez-Flota and De Luca 1998). JA also induced vindoline accumulation when applied to very young seedlings (Liscombe et al. 2010b; Vázquez-Flota et al. 2004) or multiple shoot cultures (Hernández-Domínguez et al. 2004; Vázquez-Flota et al. 2009), but not when applied to older seedlings or mature plants (El-Sayed and Verpoorte 2004, 2005; Guirimand et al. 2010; Pan et al. 2010; Y. jie Pan et al. 2018). When caterpillars fed on mature *C. roseus* plants, inducing endogenous JA synthesis, upstream strictosidine levels increased rapidly in nearby mature leaves within a day, but vindoline and catharanthine levels only increased in young or emerging leaves a week after feeding (Bernonville et al. 2017). The transcription factor that integrates the signaling between light, JA, and development in these examples has not been identified. One potential mechanism integrating these signals is through DELLA transcription factors.

DELLAs are known master negative regulators of gibberellic acid (GA) signaling involved in development but they also mediate cross-talk between multiple signaling pathways, including light and JA signaling, and leaf development. DELLAs are a member of the GRAS protein family, named after the three *Arabidopsis thaliana* genes used to define this family: GIBBERELLIC-ACID INSENSITIVE (**G**AI), REPRESSOR of GAI (**R**G**A**), and SCARECROW (**S**CR) (Itoh et al. 2002; Pysh et al. 1999). Like other GRAS proteins, DELLAs have a conserved C-terminal domain, which is responsible for homo- and hetero-dimerization (de Lucas et al. 2008; Gallego-Bartolomé et al. 2012; Itoh et al. 2002; Jiang et al. 2022; Marín-de la Rosa et al. 2015; Pysh et al. 1999; Yoshida et al. 2014a). As a result, DELLAs can interact with over 300 transcription factors and mediate cross-talk between multiple signaling pathways (Blanco-Touriñán, Serrano-Mislata, et al., 2020b). What distinguishes DELLAs from other GRAS proteins is the presence of “DELLA” and “TVHYNP” motifs in their N-terminus (Itoh et al. 2002; Pysh et al. 1999), which bind to GA and have transactivation activity (Hirano et al. 2012). Thus, DELLAs can bind through its C-terminal domain and act either as co-repressors by disrupting the activity of transcription factor partners (de Lucas et al. 2008; Feng et al. 2008; Gallego-Bartolomé et al. 2010; Hou et al. 2010; Leone et al. 2014; Li et al. 2016; Panda et al. 2022; Wild et al. 2012; Xie et al. 2016; Yang et al. 2012; Yu et al. 2012) or as co-activators by lending its N-terminal activation domain to a DNA-binding partner (Aoyanagi et al. 2020; Fukazawa et al. 2014, 2021; Hernández-García et al. 2024; Jiang et al. 2022; Marín-de la Rosa et al. 2015; Yoshida et al. 2014b).

DELLA protein levels are regulated in part by GIBBERELLIN-INSENSITIVE DWARF1 (GID1). In this mechanism, GA forms a complex with GID1 that promotes DELLA’s degradation (Alyssa et al. 2001; Murase et al. 2008; Phokas and Coates 2021) and relieves DELLA’s repression of GA-regulated genes. Mutations or truncations in the N-terminal motifs disrupt the interaction with GA and thereby with GID1, leading to constitutively elevated levels of DELLA proteins and a semi-dwarf phenotype (Alyssa et al. 2001; Boss and Thomas 2002; Cassani et al. 2009; Hirano et al. 2010; Muangprom et al. 2005; Peng et al. 1997; Winkler and Freeling 1994).

In addition to their central role in GA signaling described above, DELLAs are involved in light signaling, positively contributing to photomorphogenesis in seedlings (Achard et al. 2007) and inhibiting shade avoidance responses in mature plants (Djakovic-Petrovic et al. 2007). Specifically, DELLAs mediate this activity by binding key light signaling factors such as PIFs (de Lucas et al. 2008; Feng et al. 2008; Gallego-Bartolomé et al. 2010; Li et al. 2016), CONSTITUTIVELY PHOTOMORPHOGENIC1 (COP1) (Blanco-Touriñán et al. 2020a; Lee et al. 2022), SUPPRESSOR OF PHYA-105 1 (SPA1), and cryptochrome 1 (CRY1) (Xu et al. 2021; Zhong et al. 2021). For example, in the dark or shade, the COP1 and SPA1 complex bind and ubiquitinate DELLAs, signaling for their degradation (Blanco-Touriñán et al. 2020a; Lee et al. 2022). In the light, COP1 relocates out of the nucleus and DELLA proteins are stabilized. Additionally, the interaction between DELLA and GID1 is interrupted in the light due to a reduction of active GA levels (Kamiya and García-Martínez 1999; Reid et al. 2002) and an interaction between DELLAs and the blue light receptor CRY1 (Xu et al. 2021; Zhong et al. 2021), which further stabilizes DELLAs. Once stabilized in the light, DELLAs can bind and inhibit the activity of PIFs (de Lucas et al. 2008; Feng et al. 2008; Gallego-Bartolomé et al. 2010; Li et al. 2016). Certain PIFs act as repressors of both photomorphogenesis (Castillon et al. 2007) and vindoline biosynthesis (Liu et al. 2019); thus, we hypothesize that the repression of PIFs by DELLAs is what leads to the activation of both photomorphogenesis and vindoline biosynthesis, which coincide in the literature (Aerts 1992; Schröder et al. 1999; Vázquez-Flota et al. 2000; Vazquez-Flota and De Luca 1998; Yu et al. 2018a).

Besides their involvement in light signaling, DELLAs also positively contribute to JA-mediated defense signaling through their interaction and repression of JASMONATE ZIM DOMAIN (JAZ) proteins (Hou et al. 2010; Leone et al. 2014; Panda et al. 2022; Wild et al. 2012; Xie et al. 2016; Yang et al. 2012). JAZs are repressors of JA-mediated defense signaling and TIA biosynthesis due to their interaction and inhibition of MYC2 (Chini et al. 2007). In response to necrotrophic pathogen attack or herbivory, plants synthesize JA, which signals for the degradation of JAZ proteins (Thines et al. 2007). This degradation relieves the repression of MYC2, which activates defense-associated genes and TIA biosynthesis (Kazan and Manners 2013; Patra et al. 2018; Schweizer et al. 2018; Wasternack and Hause 2013; Zhang et al. 2011). The degradation of JAZs also frees DELLAs to bind and inhibit PIFs, leading to reduced growth in the presence of JA (Yang et al. 2012). JA additionally decreases active GA biosynthesis, increasing DELLA levels during pathogen attack (Heinrich et al. 2013). Under light, the JA response is amplified since DELLAs are stabilized with light and bind and sequester remaining JAZs (Leone et al. 2014); this is a potential explanation for the JA-induced expression of the vindoline pathway gene *D4H* in seedlings only in the presence of light (F. A. Vázquez-Flota and De Luca 1998) when DELLAs are stabilized and present.

In addition to their role in light and JA signaling, DELLAs are also involved in young leaf development. DELLAs inhibit the vegetative phase transition from juvenile to adult leaves by binding and inhibiting SQUAMOSA PROMOTER BINDING–LIKE (SPL) transcription factors (Yu et al. 2012); DELLAs also inhibit leaf senescence by binding and inhibiting certain WRKY transcription factors (Chen et al. 2014, 2017; Lei et al. 2020; Zhang et al. 2018, 2021). Thus, DELLAs promote a juvenile leaf state, the developmental condition where the highest vindoline levels are observed (Besseau et al. 2013; Góngora-Castillo et al. 2012; Mall et al. 2019; Qu et al. 2015; St-Pierre et al. 1998, 1999).

In short, DELLAs positively regulate light-signaling, positively regulate JA-signaling, and inhibit leaf aging. Similarly, vindoline pathway genes are expressed most highly in light- and JA-exposed young leaves (Aerts et al. 1994; Besseau et al. 2013; Cole-Osborn et al. 2024a; Góngora-Castillo et al. 2012; Hernández-Domínguez et al. 2004; Liscombe et al. 2010a; Liu et al. 2019; Mall et al. 2019; Qu et al. 2015; Raina et al. 2012; Schröder et al. 1999; St-Pierre et al. 1998, 1999; van der Fits & Memelink, 2000b; Vázquez-Flota and De Luca 1998; Vazquez-Flota and De Luca 1998; Wang et al. 2010; Wei 2010; Yu et al. 2018b; Zhou et al. 2015), leading us to hypothesize that DELLAs may positively regulate vindoline biosynthesis. This hypothesis is further supported by studies which showed that GA application to *C. roseus*, which results in DELLA degradation by GID1, decreased vindoline content (El-Sayed and Verpoorte 2004; Jaleel et al. 2009; Pan et al. 2010). In contrast, dwarf varieties of *C. roseus*, potentially attributed to gain-of-function (GOF) DELLA mutations, consistently contained high vindoline levels (Chung et al. 2011; Heijden et al. 2005; Mall et al. 2021).

In this paper, we explore the hypothesis that DELLAs positively contribute to the regulation of vindoline biosynthesis. We first identified two *DELLA* and two *GID1* genes in *C. roseus* using BLAST and protein alignment. Next, we experimentally confirmed the identification of the *DELLA* and *GID1* genes in *C. roseus* using virus-induced gene silencing, observing the expected growth-related phenotypes. Due to limited and complex methods for constructing fully transgenic *C. roseus* plants, we employed transient methods aimed at increasing DELLA protein levels (virus-induced gene silencing of GID1 genes, application of the GA biosynthesis-inhibitor paclobutrazol, and transient overexpression of GA-insensitive truncated DELLAs or GOF DELLAs) to test this hypothesis and investigate their effect on the expression of vindoline pathway genes and on alkaloid accumulation. Our conclusions are limited by variability in the transient methods employed, but when considered together, they provide weak to moderate evidence (Muff et al. 2022) suggesting that DELLAs can positively influence TIA biosynthesis under certain conditions.

We discuss our results using evidence-based language, which uses the p-value as a tool to discuss a continuous spectrum of evidence (i.e. no evidence, weak, moderate, or strong evidence) rather than as a binary classifier of statistical significance (Muff et al. 2022). Our results do not unequivocally prove that DELLAs directly regulate vindoline biosynthesis in *C. roseus*. However, they provide some evidence suggesting that increasing DELLA protein levels could lead to increased vindoline biosynthesis. Given the medicinal importance of *C. roseus* and its terpenoid indole alkaloids, these results encourage further inquiries into the role of DELLA proteins in TIA regulation and as a potential strategy to increase the supply of these critical chemotherapeutics.

## Materials and methods

### Identification of CrDELLA1, CrDELLA2, CrGID1a, and CrGID1b

To identify DELLA proteins in *C. roseus*, the N-terminal region containing the DELLA and TVHYNP motifs from the *A. thaliana* RGA protein (DDELLAVLGYKVRSSEMAEVALKLEQLETMMSNVQEDGLSHLATDTVHYNPSELYSWLDNMLSELNPPPLP) was used in a Protein Basic Alignment Search Tool (BLASTP) search in the *C. roseus* version 2 translated transcriptome with default parameters (BLOSUM62 Matrix, gap cost existence = 11, gap cost extension = 1) (Franke et al. 2019). To determine which of the sequences truly featured the DELLA domain, the two putative CrDELLA sequences identified by this search (CrDELLA1 = CRO_T106013 and CrDELLA2 = CRO_T106004) and the two next closest homologs in *C. roseus* (CRO_T119352 and CRO_T119350) were aligned against *A. thaliana* and *O. sativa* DELLA proteins using ClustalW with default parameters (Thompson et al. 1994) in the multiple sequence alignment (msa) R package (Bodenhofer et al. 2015). Amino acid sequences used in the alignment were downloaded from UniProt: AtRGA (*Arabidopsis thaliana*, Q9SLH3), AtGAI (*Arabidopsis thaliana*, Q9LQT8), AtRGL1 (*Arabidopsis thaliana*, Q9C8Y3), AtRGL2 (*Arabidopsis thaliana*, Q8GXW1), AtRGL3 (*Arabidopsis thaliana*, Q9LF53), OsSLR1 (*Oryza sativa*, Q7G7J6). Domain annotations on the amino acid alignment are defined according to Itoh et al. (2002) and Pysh et al. (1999).

To identify GID1 proteins in *C. roseus*, we performed a BLASTP search with the three *A. thaliana* GID1 amino acid sequences: AtGID1a (Q9MAA7), AtGID1b (Q9LYC1), AtGID1c (Q940G6). The two putative GID1 sequences identified by this search (CrGID1a = CRO_T105824 and CrGID1b = CRO_T119046) and the next closest homolog (CRO_T115705) were aligned against *A. thaliana* and *O. sativa* GID1 proteins using ClustalW with default parameters (Thompson et al. 1994) in the msa R package (Bodenhofer et al. 2015). Amino acid sequences used in the alignment were downloaded from UniProt: AtGID1a (*Arabidopsis thaliana*, Q9MAA7), AtGID1b (*Arabidopsis thaliana*, Q9LYC1), AtGID1c (*Arabidopsis thaliana*, Q940G6), OsGID1 (*Oryza sativa*, Q6L545). Annotations on the amino acid alignment are defined according to Shimada et al. (2008) and Gazara et al. (2018).

### Cloning: amplification of coding sequences and cloning for Y2H assay

Coding sequences for *CrDELLA1* (CRO_T106013), *CrDELLA2* (CRO_T106004), *CrPIF4/5* (KR703668.1, CRO_T136917)(Liu et al. 2019), and *CrJAZ1∆1–84* (FJ040204.1, CRO_T107113) (Patra et al. 2018; Zhang 2008) were amplified from *C. roseus* var. Little Bright Eye cDNA using Phusion High-Fidelity DNA Polymerase (New England BioLabs) and Gateway-compatible primers (Table S1). *CrDELLA1* and *CrDELLA2* were amplified from cDNA prepared from a pool of *C. roseus* tissues (siliques, flower buds, flowers, stems, leaves, and roots). The expected band size for each coding sequence (CDS) was cut out of an agarose gel and purified using the Zymoclean Gel DNA Recovery kit (Zymo Research). Purified PCR products were cloned into the entry plasmid pDONR^TM^221 using the Gateway^®^ BP Clonase™ II Enzyme Mix, sequence confirmed, and then cloned into the Yeast-2-Hybrid Gateway prey vector, pDEST^TM^22, or bait vector, pDEST^TM^32, using the Gateway^®^ LR Clonase™ II Enzyme Mix. To obtain the truncated *CrDELLA1∆1-209* in the pDEST^TM^32 bait plasmid, we used round-the-horn PCR on the entry vector, pDONR^TM^221-*CrDELLA1*, with primers targeting amino acids 1 to 209 (Table S1).

### Yeast two-hybrid assay

Yeast Two-Hybrid (Y2H) assays were conducted using the ProQuest™ Two-Hybrid System from Invitrogen. *CrJAZ1∆1–84* and *CrPIF4/5* coding sequences were cloned into the pDEST^TM^22 backbone containing the GAL4 Activation Domain (prey), and *CrDELLA1∆1-209* was cloned into the pDEST^TM^32 backbone containing the GAL4 DNA binding domain (bait). Yeast strain MaV203 was co-transformed with a prey and bait plasmid using the LiAc/SS carrier DNA/PEG method (Gietz and Schiestl 2007) and plated on synthetic complete (SC) media lacking leucine (to select for pDEST^TM^32), and tryptophan (to select for pDEST^TM^22) (SC-L-T media: 27 g/L dropout base medium, MP Biomedicals; 1.57 g/L synthetic complete amino acid mixture lacking histidine, leucine, tryptophan, and uracil, Sunrise Science Products; 100 mg/L adenine hemisulfate; 85.6 mg/L histidine; 85.6 mg/L uracil; 20 g/L agar; and pH adjusted to 5.8–5.9). Colonies were screened for positive interaction by plating on SC-L-T-H selection media (SC-L-T media without histidine) containing 50 mM 3-amino-1,2,4-triazole (3-AT, added after autoclaving).

### Cloning for VIGS experiments

For silencing experiments, 300 bp fragments targeting *CrDELLA1*, *CrDELLA2*, *CrGID1a*, and *CrGID1b* were designed using the Sol Genomics Network (SGN) VIGS tool (Fernandez-Pozo et al. 2015) (n-mer = 21–23, mismatches = 1), which minimized off-targets in the *C. roseus* version 2 transcriptome (Franke et al. 2019). Fragments were amplified from *C. roseus* leaf cDNA (*CrGID1a* and *CrGID1b*) or from a previously cloned plasmid (*CrDELLA1* and *CrDELLA2*) using primers listed in Table S1 and were cloned into pTRV2-GG (Addgene plasmid #105349). Plasmids targeting the Magnesium Chelatase subunit H (*ChlH*) and green fluorescent protein (*GFP)* for silencing were cloned previously (Cole-Osborn et al. 2024).

### Virus-induced gene silencing

Virus-induced gene silencing (VIGS) was performed as described previously (Cole-Osborn et al. 2024). *C. roseus* var. Little Bright Eye seeds (0.4 g, NESeeds) were sterilized and spread on full-strength Gamborg’s media (3.1 g/L Gamborg’s basal salts, 1X Gamborg’s vitamins, and 6% micropropagation agar type 1, Phytotechnology Laboratory) inside a sterile Magenta™ Plant Culture Box for germination. Seeds were germinated in the dark at 25–27˚C for about 7 days, and then were transferred to a 16 h light / 8 h dark photoperiod (red and blue LED lights, about 80 µmol m^− 2^ s^− 1^) for at least two days. Once seedlings had undergone photomorphogenesis, they were planted in soil (Miracle-Gro) and grown until two true leaves appeared (about 4–6 weeks).

At this time, they were infected with *Agrobacterium tumefaciens* GV3101 (pMP90) according to the pinch-wounding method (Liscombe and O’Connor 2011). *A. tumefaciens* containing pTRV2 or pTRV1 plasmids were combined in a 1:1 ratio (OD_600_ of each strain = 2–4). Modified tweezers were dipped into the *A. tumefaciens* solution and the plant was pinched three times in the highest internode beneath the shoot apical meristem (dipping into the solution between each pinch). After infection, plants were kept in the dark for two days before being placed back into a 16 h light / 8 h dark photoperiod. Plants were grown until two pairs of leaves emerged after the VIGS procedure was carried out, and *ChLH*-silenced positive control plants exhibited yellow leaves (about 2–3 weeks). In addition, plants were confirmed to be *CrDELLA-* or *CrGID1-*silenced using qPCR. For each of the two leaf pairs that emerged after the VIGS procedures, one leaf or half of a leaf (cut lengthwise) was harvested for RNA extraction and one leaf or half of a leaf was harvested for alkaloid analysis. Leaves were flash-frozen in liquid nitrogen, and stored at -80˚C until downstream procedures.

### Plant phenotype measurements

Leaf lengths and widths were measured with a ruler for two experimental repeats. For a third experimental repeat, the leaves were laid flat next to a ruler and photographed; lengths and widths were measured from photographs using ImageJ. Lengths were measured from the base of the petiole to the tip of the leaf. Widths were measured at the widest part of the leaf.

Seedlings treated with paclobutrazol (PAC) were photographed next to a ruler and lengths from the tip of the root to the tip of the cotyledon were measured with ImageJ.

### RNA extraction and quantitative PCR

Gene expression levels were monitored using quantitative real-time PCR (qRT-PCR) as described previously (Cole-Osborn et al. 2024), with primers listed in Table S1. Tissue was flash frozen in liquid nitrogen, and then crushed with glass beads in a Mini-BeadBeater-16 (Biospec). RNA was extracted with RNAzol-RT (Molecular Research Center) and the Direct-zol RNA Miniprep Plus Kit (Zymo Research). cDNA was synthesized using either the SuperScript II First-Strand Synthesis System (Invitrogen) or the LunaScript RT SuperMix Kit (New England Biolabs). cDNA was diluted 1:4, and 1 µL was used in a 10 µL reaction with SYBR Green ROX qPCR Master Mix (Qiagen or ABClonal) and 300 nM primers on the MX3000P (Agilent) or CFX96 (Bio-Rad) qPCR instrument (Agilent). In the data analysis, Ct values for each biological replicate were calculated as the average of two technical replicates. Transcript levels were normalized to the housekeeping gene, *SAND* (Pollier et al. 2014), and fold changes relative to the negative control condition were calculated according to the 2^−∆∆Ct^ method (Livak and Schmittgen 2001).

### Alkaloid extraction

Alkaloids were extracted as described previously (Cole-Osborn et al. 2024). Tissue was weighed, flash frozen in liquid nitrogen, and then crushed with glass beads in a Mini-BeadBeater-16 (Biospec). Alkaloids in the crushed leaves were extracted with methanol (1 mL) 3 times; the methanol extract was pooled and concentrated under vacuum using a Speedvac. Prior to analysis, samples were redissolved in 20 mL methanol per gram of fresh weight and refrigerated at 4˚C for at least 4 h and centrifuged to remove particulates. Finally, extract was diluted 1:50 in 50% methanol/50% water.

### Alkaloid quantification with HPLC-MS-MS

Alkaloid quantification was performed at the Mass Spectrometry Facility at Northeastern University, as described previously (Cole-Osborn et al. 2024). Alkaloids were separated with a Phenomenex Luna Omega LC column (1.6 μm C18 100 A°, 2.1 × 50 mm) on the Thermo Scientific™ Vanquish HPLC system. The mobile phase consisted of 0.1% formic acid in water (solvent A) and 0.1% in acetonitrile (solvent B). Using an injection volume of 1 µL, alkaloids were separated using a gradient from 15 to 31% solvent B at a flow rate of 0.3 mL/min and a column temperature of 35 °C.

The compounds were detected on a Tandem HRMS orbitrap mass analyzer (Thermo Scientific Exploris 240) coupled to an electrospray ionization (H-ESI) source in the positive mode with typical settings (Cole-Osborn et al. 2024). Quantitative analysis was performed in the full scan MS with data-dependent tandem mass spectrometry. The parent ion and the confirmatory fragment ion at the optimal collision energies (CE) of each alkaloid was ajmaline 327.21→ 158.10 (CE 57), catharanthine 337.19 → 144.08 (CE 31.5), tabersonine 337.19 → 305.17 (CE 31.5), serpentine 349.16 → 263.08 (CE 46), ajmalicine 353.19 → 144.08 (CE 30), vindoline 457.23 → 188.11 (CE 34), vinblastine 811.42 → 751.4 (CE 51), and vincristine 825.40 → 765.38 (CE 52). The data processing and area under the curve of the extracted ion chromatogram (XIC) was performed on Thermo Scientific Xcalibur Version 4.5.474.0.

The extraction method resulted in > 95% of total alkaloids extracted from 50 mg dry weight leaf tissue (~ 500 mg fresh weight) with the percent recovery measured at 100 +/- 10%. The linear range of the quantified alkaloids was validated with a calibration curve of standards prepared in solvent.

### Paclobutrazol treatment of seedlings

*C. roseus* var. Little Bright Eye seeds (NESeeds, 0.8 g) were sterilized by submersion in 4% Plant Preservative Mixture (PPM) for 18 h in the dark. The PPM was then decanted, and the seeds were spread on full-strength Gamborg’s media (3.1 g/L Gamborg’s basal salts, 1X Gamborg’s vitamins, and 6% micropropagation agar type 1, Phytotechnology Laboratory) inside a sterile Magenta™ Plant Culture Box (Sigma) for germination. Seeds were germinated in the dark at 27˚C for 5 days until the radicle of the seedling had just emerged. A 100 mM stock solution of paclobutrazol (PAC, PhytoTechnology Laboratories) was prepared with DMSO as the solvent, filter-sterilized, and stored at -20˚C until use. A final concentration of 1 µM PAC or an equivalent amount of DMSO (mock) was added to Gamborg’s media after autoclaving. After germination, seedlings were sterilely transferred to a new Magenta™ Box containing Gamborg’s media containing 1 µM PAC or DMSO. Seedlings were maintained in the dark at 27˚C for 4 days, and then were harvested (3 whole seedlings in a 2 mL screw cap tube containing ten 3 mm glass beads for each biological replicate), flash-frozen in liquid nitrogen and stored at -80˚C until ready for RNA extraction and qPCR analysis.

### Cloning for overexpression experiments: *CrDELLA* overexpression plasmids and vindoline pathway reporter plasmids

To overexpress full-length *CrDELLA1*, the coding sequence was amplified from a previously cloned plasmid using Golden-Gate compatible primers (Table S1). The amplified PCR product was gel extracted, cut with BpiI, and ligated into pICH41308 (level zero CDS1 backbone). Full-length *CrDELLA1* was then cloned into a transcriptional unit in pICH47811 (level 1 reverse position 2 vector backbone), along with the Cauliflower mosaic virus 2 × 35 S promoter, Tobacco Mosaic Virus (*TMV*) omega 5’UTR, and *Agrobacterium tumefaciensMAS *(*AtuMAS) *terminator. This transcriptional unit was moved into the pSB90 backbone (Addgene plasmid #123187), which includes the right and left borders required for *Agrobacterium*-mediated transfer into plant cells, and a mutated *VirG* gene to enhance *Agrobacterium *virulence (Mortensen et al. 2019). pSB161 (Mortensen et al. 2019) was used as a negative control, which contains *Beta-glucuronidase* (*GUS*) with an intron under control of the 2 × 35 S promoter.

To overexpress *CrDELLA1∆1-112* or *CrDELLA2∆1-113*, the truncated coding sequences were amplified from a previously cloned plasmid using Golden-Gate compatible primers (Table S1). The amplified PCR product was gel extracted, cut with BpiI, and ligated into pICH41308 (level zero CDS1 backbone). The truncated coding sequences were then cloned into a transcriptional unit in pICH47732 (level 1 forward position 1 vector backbone), containing the *A. thaliana Ubiquitin 10* promoter and *AtuMAS *terminator. This transcriptional unit was moved into the pSB90 backbone.

Vindoline pathway promoters and 5’UTRs were previously amplified, sequence confirmed, and cloned into level zero promoter + 5’UTR backbones (Cole-Osborn et al. 2024). Promoters were moved into a transcriptional unit in pICH47822 (level 1 reverse position 3 vector) with the promoter + 5’UTR driving the firefly luciferase gene (containing plant-specific introns (Mortensen et al. 2019) with the *AtuOCS* terminator. This transcriptional unit was moved into the pSB90 backbone with a second transcriptional unit that included the *Renilla* luciferase gene (containing plant-specific introns (Mortensen et al. 2019) under control of the *AtuNOS* promoter, *TMV* omega 5’UTR, and *AtuNOS* terminator in Forward position 1. Vindoline pathway reporter plasmids were deposited at Addgene (Accessions: 203896–203902).

All primers used for cloning are listed in Table S1. Sequences were confirmed after every PCR amplification using Sanger Sequencing at Genewiz^®^. Final plasmids were confirmed with a restriction enzyme digest and visualized with agarose gel electrophoresis. All L2 plasmids were electroporated into *Agrobacterium tumefaciens* GV3101 (pMP90).

### Efficient *Agro*-mediated seedling infiltration (EASI) and dual-luciferase assay

For promoter transactivation experiments, seedlings were transformed according to the efficient *Agro*-mediated seedling infiltration (EASI) method (Cole-Osborn, Meehan, et al., 2024; Mortensen et al. 2019, 2022) with *A. tumefaciens* strains containing either an effector plasmid or a reporter plasmid. The effector plasmid encoded *CrDELLA1∆1-112*, *CrDELLA2∆1-113*,* or* beta-glucuronidase (*GUS*, negative control) driven by a constitutive *A. thaliana Ubiquitin10* promoter. The reporter plasmid encoded a vindoline pathway promoter of interest driving the expression of intron-containing firefly luciferase (*FLUC*) gene and a constitutive *Atu*NOS promoter driving expression of intron-containing *Renilla* luciferase (*RLUC*) gene for normalizing differences in transformation efficiency. *A. tumefaciens* strains encoding the effector or reporter plasmids were mixed in a 1:1 ratio at a final OD_600_ of 0.4 (OD_600_ = 0.2 for each strain). After infiltration, seedlings were kept in the dark for 2 days, and then moved to continuous light (red and blue LED lights) at room temperature (~ 22˚C) for 24 h prior to harvest.

For RNA extraction, cotyledons were isolated from 5 seedlings and pooled for each biological replicate (in a 2 mL screw cap tube containing ten 3 mm glass beads). Samples were flash-frozen in liquid nitrogen and stored at -80˚C until needed. For dual-luciferase assays, two whole seedlings were pooled for each biological replicate (in a 1.5 mL screw cap tube containing three 3 mm glass beads) and protein was extracted and used in a dual-luciferase assay, as described previously (Mortensen et al. 2019). Seedlings transformed with intron-containing *GUS* overexpression plasmids underwent histochemical staining to confirm transformation success prior to the luciferase assay, as described previously (Mortensen et al. 2019).

### Statistical analysis

When multiple experimental repeats were performed (Figs. 4, 6 and 8; Figure S6, S8), “experimental repeat” was included as a factor in a two-way ANOVA / two-factor linear model for statistical analysis. The other factor was either *CrDELLA*-silencing, *CrGID1*-silencing, or *PAC* treatment. A full factorial standard least squares linear model was fitted for each dependent variable (leaf length: width ratio, gene expression, or alkaloid levels), and F-tests were performed in JMP Pro 15 to test the impact of each factor to the model. The normality of residuals was checked with an Anderson-Darling Goodness-of-Fit Test; if linear values were not normally distributed, values were log-transformed, and residuals were again checked for normality. The resulting p-values from the effect tests were adjusted for false discovery rate (FDR) using a two-stage linear step-up procedure of Benjamini, Krieger and Yekutieli (Q = 5%) in GraphPad Prism v. 9.5.1. Full two-way ANOVA results can be found in Supplemental Data 1.

**Fig. 1:**
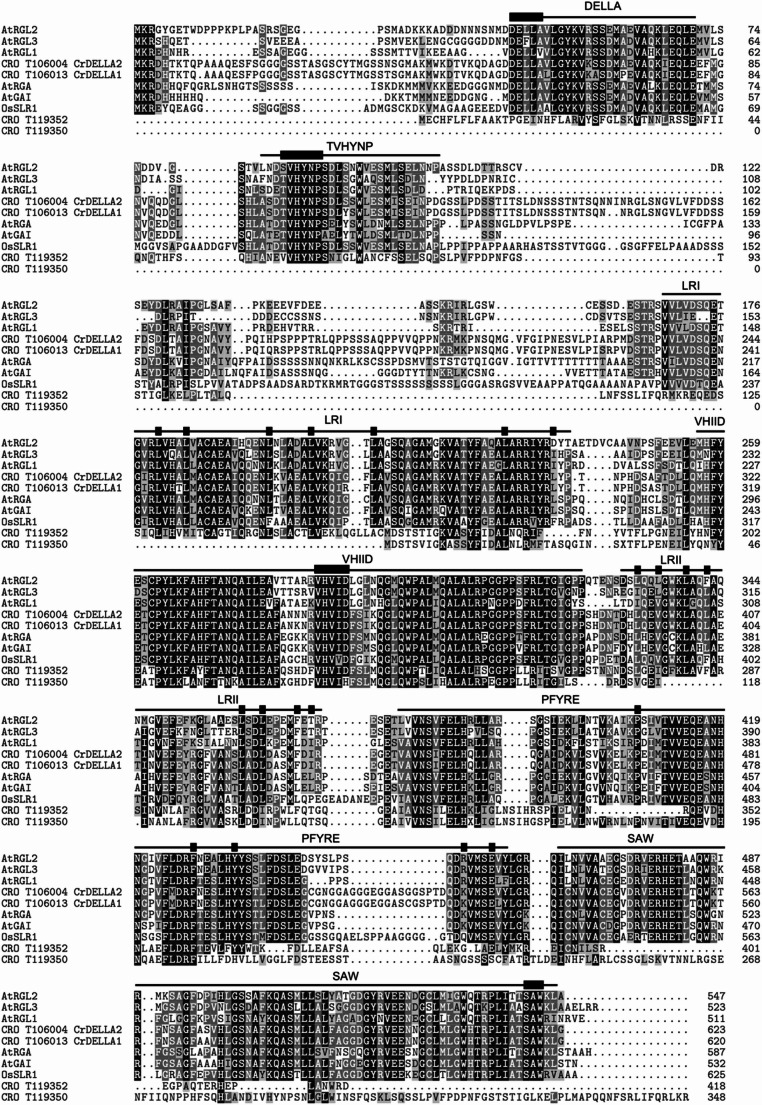
Amino acid alignment of DELLA proteins. Protein sequences were downloaded from Uniprot with the following accession numbers: AtRGA (*Arabidopsis thaliana*, Q9SLH3), AtGAI (*Arabidopsis thaliana*, Q9LQT8), AtRGL1 (*Arabidopsis thaliana*, Q9C8Y3), AtRGL2 (*Arabidopsis thaliana*, Q8GXW1), AtRGL3 (*Arabidopsis thaliana*, Q9LF53), OsSLR1 (*Oryza sativa*, Q7G7J6). Sequences were aligned with CrDELLA1 (CRO_T106013), CrDELLA2 (CRO_T106004), and the two next closest homologs in *C. roseus*, CRO_T119352 and CRO_T119350, using ClustalW with default parameters (Thompson et al. 1994) in the msa R package (Bodenhofer et al. 2015). Black shading indicates residues with > 80% conservation, dark gray shading indicates residues with > 50% conservation, light gray shading indicates residues with similar properties, and white shading indicates non-conserved residues. Lines indicate conserved domains, and filled boxes indicate defining amino acid residues for each domain. Annotations are defined according to Pysh et al. (1999), Itoh et al. (2002), and Bolle (2004)

**Fig. 2:**
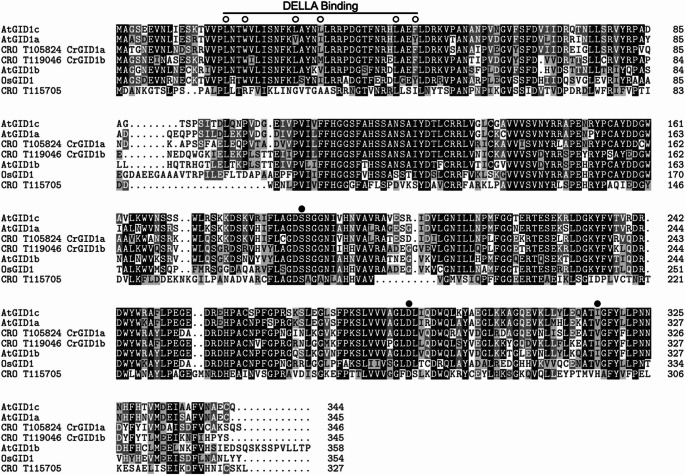
Amino acid alignment of GID1 proteins. Protein sequences were downloaded from UniProt: OsGID1 (*Oryza sativa*, Q6L545), AtGID1a (*Arabidopsis thaliana*, Q9MAA7), AtGID1b (*Arabidopsis thaliana*, Q9LYC1), AtGID1c (*Arabidopsis thaliana*, Q940G6). Sequences were aligned with CrGID1a (CRO_T105824), CrGID1b (CRO_T119046), and the next closest homolog in *C. roseus*, CRO_T115705, using ClustalW with default parameters (Thompson et al. 1994) in the msa R package (Bodenhofer et al. 2015). Black shading indicates residues with > 80% conservation, dark gray shading indicates residues with > 50% conservation, light gray shading indicates residues with similar properties, and white shading indicates non-conserved residues. Open circles are conserved residues important for binding to DELLAs. Filled circles are the “catalytic triad” SDV/I present in GID1s. Annotations are defined according to Shimada et al. (2008) and Gazara et al. (2018)

**Fig. 3:**
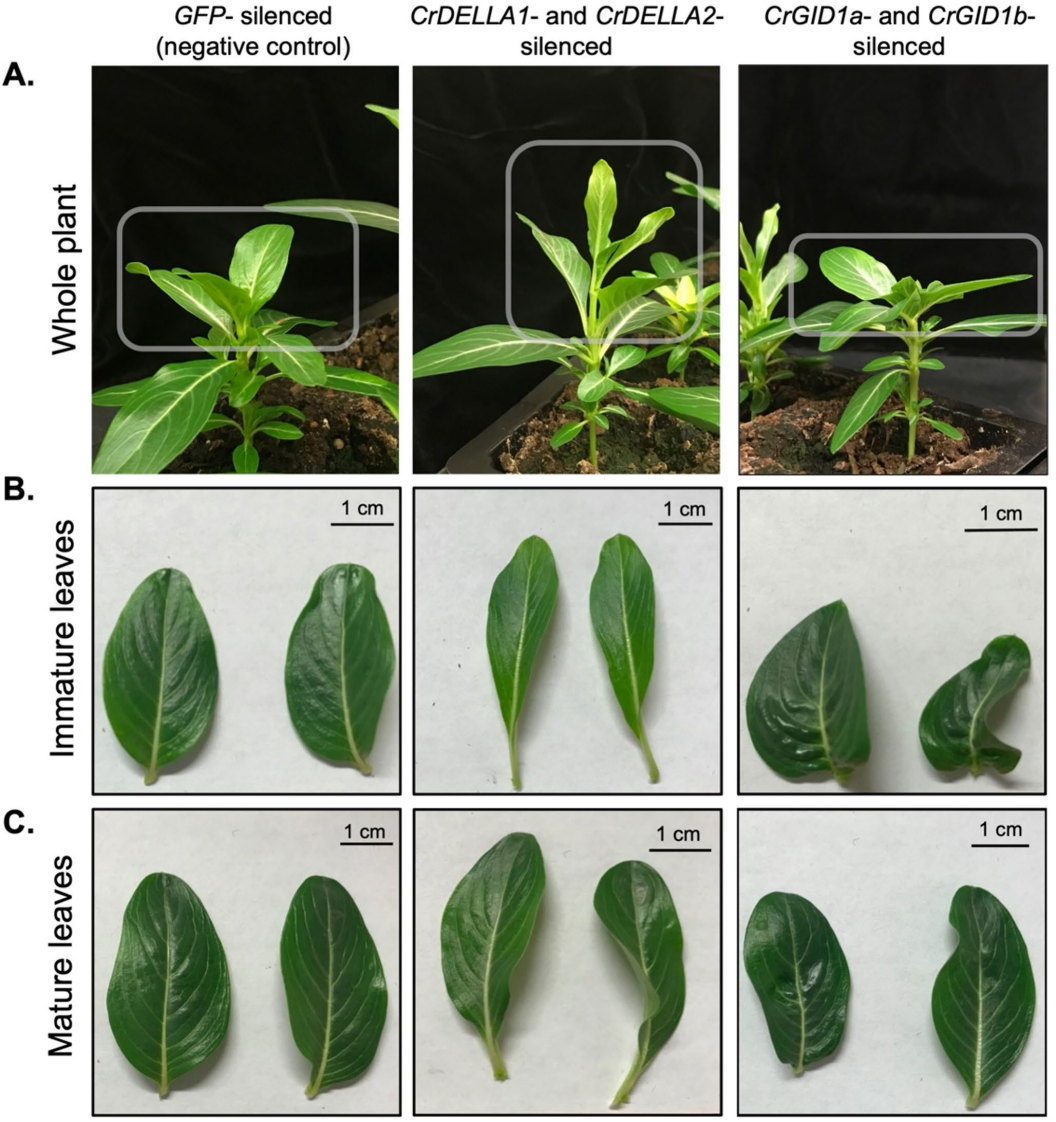
The physical phenotypes of *CrDELLA1*- and *CrDELLA2*-silenced plants and *CrGID1a*- and *CrGID1b*-silenced plants compared to *GFP*-silenced plants (negative non-targeting control). (**A**) *CrDELLA*-silenced plants generally show an elongated stem, elongated leaves, and leaves pointing upward (hyponasty). *CrGID1*-silenced plants in contrast show a shortened stem, shortened leaves, and horizontally aligned leaves (epinasty). Note that the phenotype will only be visible in the two youngest leaf pairs where VIGS silencing has occurred (highlighted with a gray box). (**B**) Isolated immature leaves from *GFP*-silenced, *CrDELLA*-silenced, and *CrGID1*-silenced plants. Immature leaves were defined as the second leaf pair to emerge after silencing. *CrDELLA-*silenced leaves were elongated. *CrGID1-*silenced leaves were short and crinkly. (**C**) Mature leaves, defined as the first leaf pair to emerge after silencing, showed a similar phenotype to immature leaves, but the phenotype was not as strong. A single plant has been shown as an example. To see the phenotype of all plants analyzed in this study, see Figure S3 and Figure S4

**Fig. 4:**
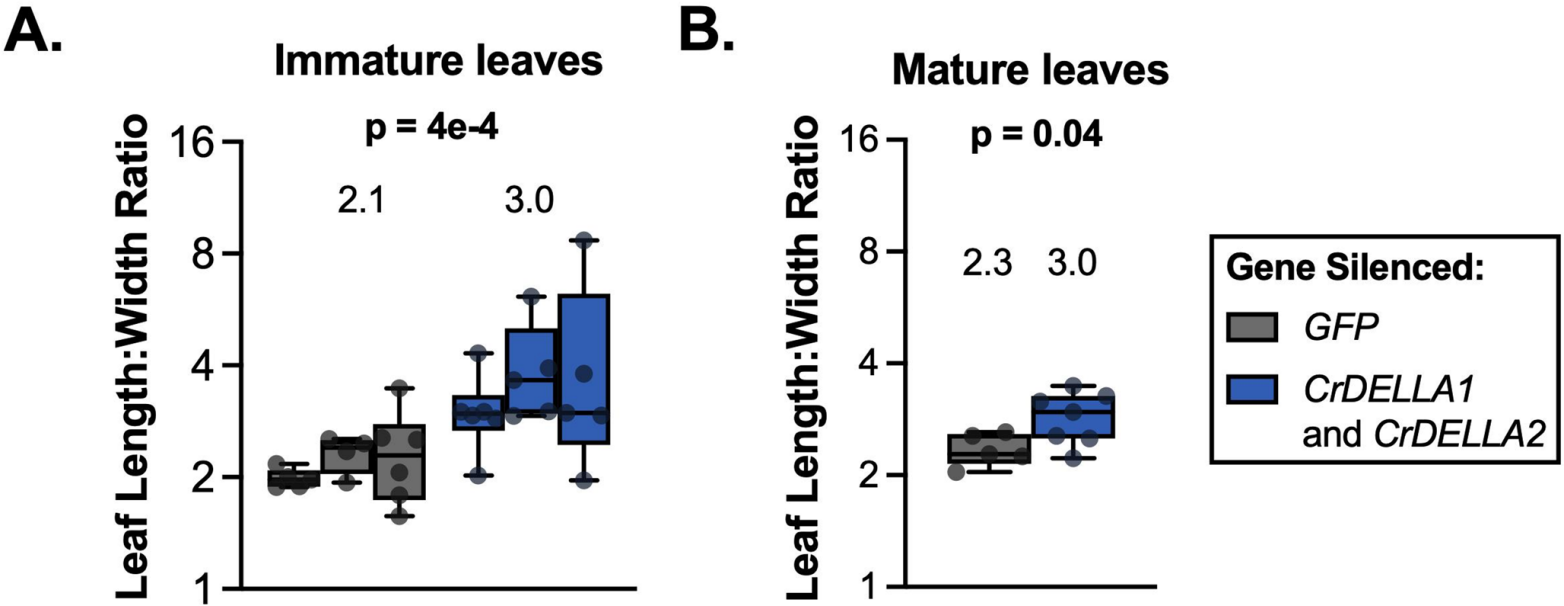
Silencing *CrDELLA1* and *CrDELLA2* causes a significant increase in the leaf length: width ratio. (**A**) Length: width ratios of immature leaves (2nd leaf pair to emerge after VIGS infection) from *CrDELLA-*silenced plants are significantly greater than *GFP*-silenced plants (negative non-targeting control). The experiment was repeated 3 times (displayed as separate but adjacent boxes; corresponds to the same 3 experiments as Fig. 6A-B). The p-value indicates the significance of the effect of gene silencing according to a two-way ANOVA on log-transformed length: width ratios, using a full-factorial model for variables “gene-silenced” and “experimental repeat”. Complete ANOVA results can be found in Supplemental Data 1. (**B**) Length: width ratios of mature leaves (1st leaf pair to emerge after VIGS infection) from *CrDELLA*-silenced plants are also significantly greater than *GFP*-silenced plants, but to a lesser extent than immature leaves. Only one experiment was analyzed for mature leaves, as explained in the subsequent section (corresponds to the same experiment as Figure S6). The p-value was calculated from an unpaired two-tailed students t-test on log-transformed length: width ratios. P-values less than 0.05 are bolded. Length was measured from the base of the petiole to the tip of the leaf. Width was measured at the widest portion of the leaf. Measurements were made with a ruler or using ImageJ. Each data point is an average of both leaves in a leaf pair from an individual plant (*n* = 5–7). For all plants, silencing was confirmed with qPCR for one leaf out of a leaf pair. Numbers above the boxes represent the median for all experiments combined. Boxes represent the 25th and 75th percentile with a line marking the median. Whiskers extend to the minimum and maximum

## Results

### Identification of DELLA proteins in *C. roseus*

To identify DELLA proteins in *C. roseus*, we performed a BLASTP search using the N-terminal region containing the DELLA and TVHYNP motifs from the *A. thaliana* RGA1 protein as a query against the *C. roseus* version 2 translated transcriptome (Franke et al. 2019). Two sequences (CRO_T106013 named CrDELLA1 and CRO_T106004 named CrDELLA2) were returned with low e-values (< 1E-30) and high sequence similarity (> 74% identity). The next closest hits from the BLASTP search (CRO_T119352 and CRO_T119350) had much lower homology (e-values < 3E-5 and < 36% identity). An amino acid alignment with *A. thaliana* and *O. sativa* DELLA proteins confirmed the presence of the N-terminal DELLA and the TVHYNP domains (involved in GA perception and transactivation) and the C-terminal leucine rich regions (LRI and LRII), VHIID, PFYRE, and SAW motifs (involved in protein-protein interaction) (Bolle 2004; Itoh et al. 2002; Pysh et al. 1999) in CrDELLA1 and CrDELLA2 but not in CRO_T119352 and CRO_T119350 (Fig. 1).

**Fig. 5:**
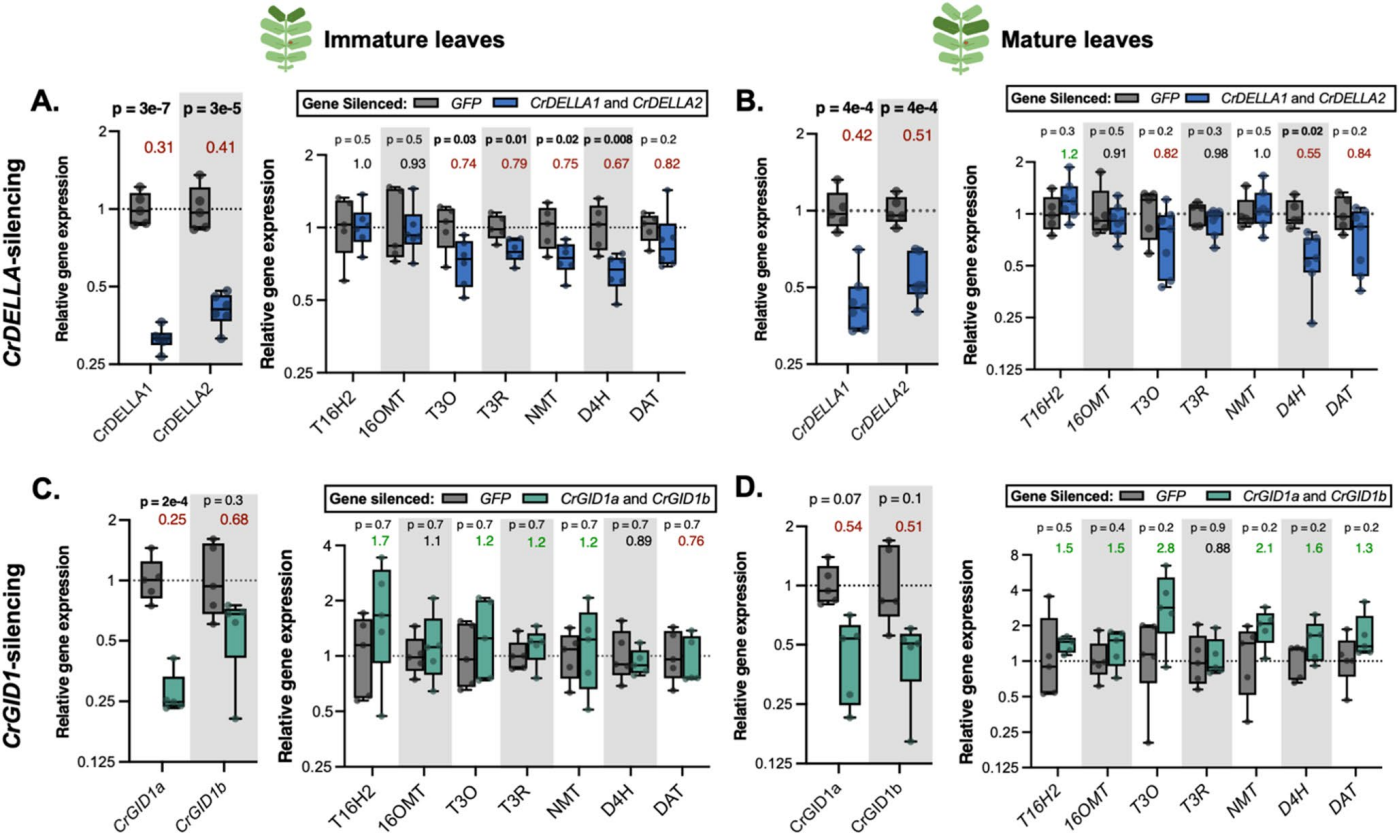
In the first experiment, silencing *CrDELLA* or *CrGID1* provided weak to moderate evidence for CrDELLAs positively regulating vindoline pathway gene expression. (**A**) In immature leaves, *CrDELLA1* and *CrDELLA2* were significantly silenced by 60–70%, and silencing significantly decreased vindoline pathway gene expression (*T3O*, *T3R*, *NMT*, *D4H*, and *DAT*) by 20–30%. (**B**) In mature leaves, *CrDELLA1* and *CrDELLA2* were significantly silenced by 50–60%, and silencing decreased vindoline pathway gene expression (*T3O*, *D4H*, and *DAT*) by 20–45%. (**C**) In immature leaves, *CrGID1a* and *CrGID1b* were silenced by 75% and 30%, respectively, and silencing did not affect vindoline pathway gene expression. (**D**) In mature leaves, *CrGID1a* and *CrGID1b* were both silenced by ~ 50%, and silencing increased vindoline pathway gene expression (*T16H2*, *16OMT*, *T3O*, *NMT*, *D4H*, and *DAT*) by 30–180%. RNA was extracted from one immature or one mature leaf for each plant (*n* = 5–7). Relative gene expression was measured with qPCR and calculated using the 2^−∆∆Ct^ method (Livak and Schmittgen 2001) relative to the non-targeting negative control condition (*GFP*-silenced plants) and normalized relative to the housekeeping gene, *SAND* (Pollier et al. 2014). Numbers above the boxes represent the median fold change relative to *GFP*-silenced plants (> 1.2 is in green, < 0.85 is in red). P-values were calculated from an unpaired two-tailed t-test on ∆∆Ct values, corrected for false discovery rate (FDR = 5%). P-values less than 0.05 are bolded. Boxes represent the 25th and 75th percentile of each experiment with a line marking the median. Whiskers extend to the minimum and maximum

Arising from a single ancestor in bryophytes, *DELLA* genes have been duplicated and lost numerous times throughout tracheophyte evolution, leading to variability in the number of *DELLA* genes between species, ranging from 1 to 14 DELLAs per species (Gallego-Bartolomé et al. 2010; Phokas and Coates 2021; Wang et al. 2020). Our identification of two *DELLA* genes in *C. roseus* is consistent with this evolutionary history.

*CrDELLA1* and *CrDELLA2* are clustered on the same genomic scaffold, about 230 kb apart (cro_v2_scaffold_123, Chr2) (Franke et al. 2019; Li et al. 2023). Using available *C. roseus* transcriptome data (Góngora-Castillo et al. 2012), we found that *CrDELLA1* is most highly expressed in roots while *CrDELLA2* is most highly expressed in mature leaves (Figure S1). We amplified and sequenced *CrDELLA1* and *CrDELLA2* from *C. roseus* var. Little Bright Eye cDNA, confirming that they were expressed and that the coding sequences predicted by CRO_T106013 and CRO_T106004 were correct. As a preliminary characterization of CrDELLA1, we performed a yeast-two hybrid (Y2H) assay, which showed that CrDELLA1 could interact with defense-signaling CrJAZ1 and light-signaling CrPIF4/5 (Figure S2); the positive interaction from the Y2H assay suggests that CrDELLA1 can mediate crosstalk between defense and light signals in *C. roseus*, similar to DELLAs in *A. thaliana* (Yang et al. 2012).

### Identification of GID1 proteins in *C. roseus*

Given the central role of GID1 in the degradation of DELLAs, we identified *GID1* genes in *C. roseus* for future viral induced gene silencing experiments as a complementary method of exploring CrDELLA function. Silencing *GID1* genes would be expected to increase DELLA protein levels hypothesized to regulate vindoline biosynthesis. GID1 is a soluble, nuclear-localized receptor that consists of a C-terminal pocket that binds specifically to active GAs and an N-terminal “lid” that stabilizes this binding and interacts with the N-terminus of DELLA proteins in a GA-dependent manner (Shimada et al. 2008; Yoshida et al. 2018). Hirano et al. proposed that this interaction between DELLA and GID1 causes a conformational change in DELLAs, facilitating DELLA’s interaction with the SKP1-CUL1-F-box (SCF) complex (Hirano et al. 2010) and its subsequent polyubiquitination and degradation by the 26 S proteosome (reviewed in (Davière and Achard 2013).

We performed a BLASTP search with the three *A. thaliana* GID1 amino acid sequences (AtGID1a, AtGID1b, AtGID1c) as queries against the *C. roseus* version 2 translated transcriptome (Franke et al. 2019). The three searches returned two top hits (CRO_T105824 and CRO_T119046) with high homology values (e-value = 0.00 and > 68% identity), which we named CrGID1a and CrGID1b, respectively. Homology values for the next closest homolog (CRO_T115705) were lower (e-value < 1E-64 and < 40% identity).

Alignment of the above three sequences together with *A. thaliana* and *O. sativa* GID1 proteins showed that CrGID1a and CrGID1b have strong conservation of the DELLA binding domain while CRO_T115705 does not (Fig. 2). This includes conservation of six hydrophobic amino acid residues that exist on the outside of the N-terminal “lid” and are critical for binding to DELLAs (Shimada et al. 2008) (Fig. 2, see open circles).

**Fig. 6:**
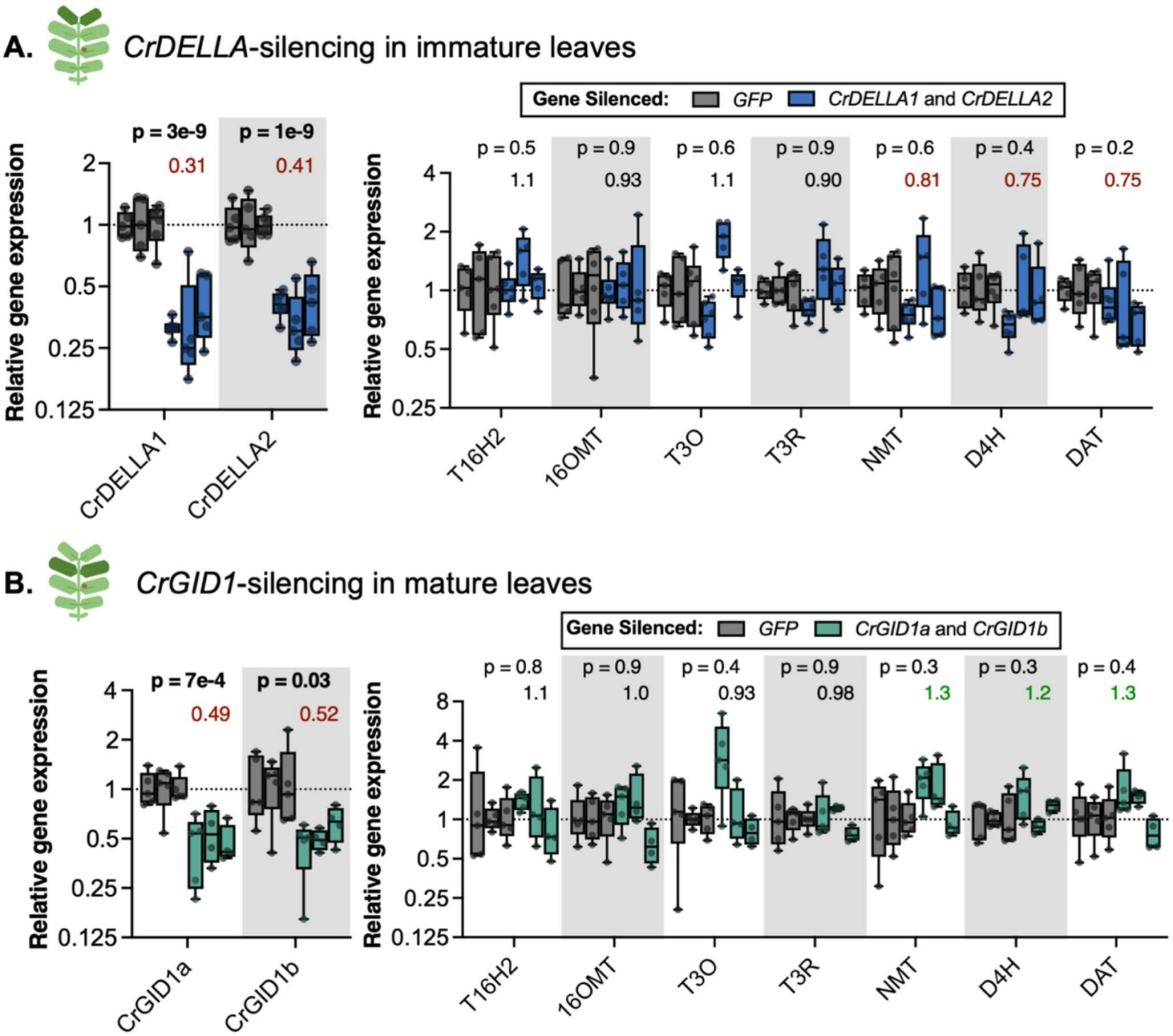
Across three experiments, there was little to no evidence that silencing *CrDELLA* or *CrGID1* affected vindoline pathway gene expression. (**A**) *CrDELLA1* and *CrDELLA2* were significantly silenced by 60–70% in *C. roseus* immature leaves, but silencing did not significantly affect vindoline pathway gene expression across three experiments. (**B**) *CrGID1a* and *CrGID1b* were significantly silenced by ~ 50% in *C. roseus* mature leaves, but silencing did not significantly affect vindoline pathway gene expression across three experiments. RNA was extracted from one immature or one mature leaf for each plant (*n* = 4–6). Plants that did not show silencing of the target genes were removed from analysis and are not shown (3 plants did not show silencing of *CrDELLA1* and *CrDELLA2*; 1 plant did not show silencing of *CrGID1a* and *CrGID1b*). The experiment was repeated 3 times (displayed as separate but adjacent boxes). Relative gene expression was measured with qPCR and calculated using the 2^−∆∆Ct^ method (Livak and Schmittgen 2001) relative to the non-targeting negative control condition (*GFP*-silenced plants) and normalized relative to the housekeeping gene, *SAND* (Pollier et al. 2014). Numbers above the boxes represent the median fold change for all of the experiments combined, relative to *GFP*-silenced plants (> 1.2 is in green, < 0.85 is in red). P-values indicate significance of the effect of gene silencing according to a two-way ANOVA on ∆∆Ct values, using a full-factorial model for variables “gene-silenced” and “experimental repeat”. P-values were corrected for false discovery rate (FDR = 5%). P-values less than 0.05 are bolded. Complete ANOVA results can be found in Supplemental Data 1. Boxes represent the 25th and 75th percentile of each experiment with a line marking the median. Whiskers extend to the minimum and maximum

GID1 proteins contain a “catalytic triad” of amino acid residues in their C-terminal pocket responsible for their strong affinity for binding to active GAs (Shimada et al. 2008). This catalytic triad consists of a serine (S), aspartic acid (D), and either valine (V) or isoleucine (I). The amino acid alignment showed that CrGID1a and CrGID1b contained either a V or I in their catalytic triads, consistent with the GID1 family, while the next closest homolog (CRO_T115705) contained a H in its catalytic triad, consistent instead with the hormone sensitive lipase (HSL) family (Shimada et al. 2008) (Fig. 2, see filled circles).

Rice and most monocots contain a single GID1 protein. Early in eudicot history, GID1 was duplicated, leading to “A-type” GID1s like AtGID1a and AtGID1c and “B-type” GID1s like AtGID1b (Yoshida et al. 2018). The presence of two GID1s in *C. roseus* may reflect this early eudicot duplication event. Available *C. roseus* transcriptome data (Góngora-Castillo et al. 2012) shows *CrGID1a* is highly expressed across tissue types whereas *CrGID1b* displays more tissue-specific expression, with high expression in flowers and stems and low expression in leaves (Figure S1).

### Silencing *CrDELLA* or *CrGID1* in *C. roseus* led to visible growth-associated phenotypes

We functionally confirmed the identified *CrDELLA* and *CrGID1* genes using virus-induced gene silencing (VIGS) and observed the expected opposite growth-related phenotypes. We constructed two pTRV2 silencing plasmids that targeted *CrDELLA1* and *CrDELLA2* for simultaneous silencing (i.e. *CrDELLA*-silenced) or *CrGID1a* and *CrGID1b* for simultaneous silencing (i.e. *CrGID1*-silenced). These plasmids were individually transformed into *Agrobacterium tumefaciens* and introduced into young *C. roseus* plants, along with *A. tumefaciens* containing the pTRV1 plasmid. The visible phenotype of our *C. roseus ChLH*-silenced plants confirmed that VIGS triggered transient silencing in the two leaf pairs that emerged after infection, as has been reported previously (Liscombe and O’Connor 2011). In addition, leaves were confirmed to be *CrDELLA*- or *CrGID1*-silenced using qPCR (Figs. 5 and 6, Figure S6).

*GID1* knockout mutants and gain-of-function *DELLA* mutants were originally identified and are well-known for their dwarf and semi-dwarf phenotype, respectively (Alyssa et al. 2001; Boss and Thomas 2002; Cassani et al. 2009; Hirano et al. 2010; Illouz-Eliaz et al. 2019; Muangprom et al. 2005; Peng et al. 1997; Winkler and Freeling 1994). In our *C. roseus* VIGS plants, we observed similar growth-related phenotypes consistent with the activity of DELLA and GID1 in the GA-signaling pathway. *CrDELLA1*- and *CrDELLA2*-silenced plants appeared elongated while *CrGID1a*- and *CrGID1b*-silenced plants appeared dwarfed compared to *GFP*-silenced (non-targeting) control plants (Fig. 3A, Figure S3, S4). These phenotypes were most clearly observed in the shapes of the two leaf pairs that experienced silencing (Fig. 3B and C).

**Fig. 7:**
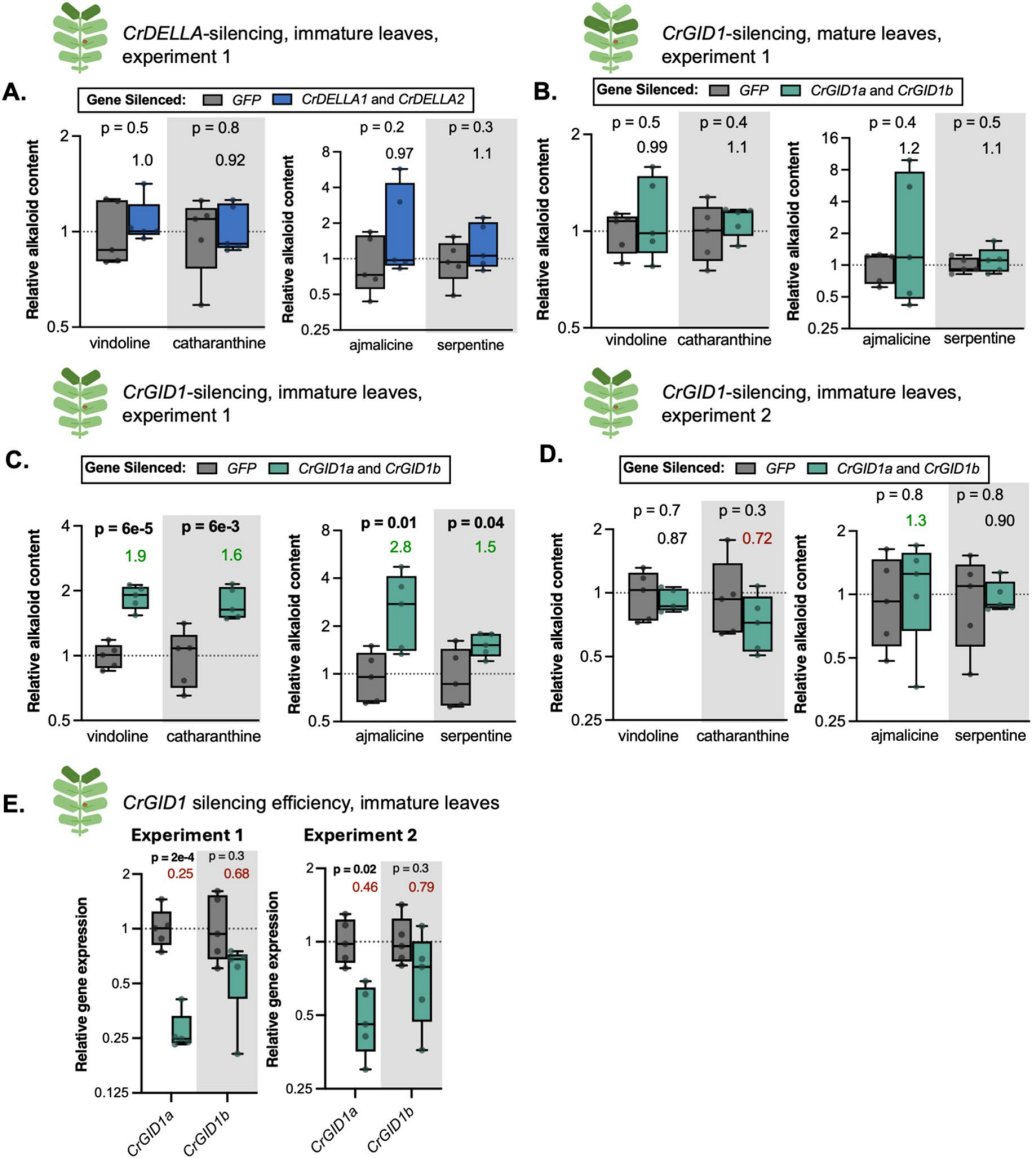
Impact of silencing *CrDELLA1* and *CrDELLA2* or *CrGID1a* and *CrGID1b* on terpenoid indole alkaloid (TIA) accumulation in *C. roseus* leaves. (**A**) In immature leaves, TIA accumulation was unaffected by *CrDELLA-*silencing. (**B**) In mature leaves, TIA accumulation was unaffected by *CrGID1*-silencing. (**C**) In immature leaves, in the first experiment, all TIAs measured (vindoline, catharanthine, ajmalicine, and serpentine) significantly increased with *CrGID1*-silencing. (**D**) However, in a second experiment, in immature leaves, TIA accumulation was unaffected by *CrGID1*-silencing. (**E**) *CrGID1a* was silenced by 75% in the first experiment, but only by 55% in the second experiment, possibly explaining discrepancies in TIA accumulation between experiments. Alkaloids were extracted from one immature or one mature leaf for each plant and analyzed using HPLC-MS-MS. Relative alkaloid contents were calculated from peak areas normalized to an internal standard, and then normalized to the *GFP*-silenced (negative control) condition for each experiment. Each replicate is from an individual plant (*n* = 4–5). Experiment 1 and experiment 2 are the same silencing experiments analyzed with qPCR (Figs. 6 and 7). Relative gene expression was measured with qPCR and calculated using the 2^−∆∆Ct^ method (Livak and Schmittgen 2001). Numbers above the boxes represent the median fold change relative to *GFP*-silenced plants (> 1.2 is in green, < 0.85 is in red). P-values were calculated from an unpaired two-tailed t-test on log-transformed values. P-values less than 0.05 are bolded. Boxes represent the 25th and 75th percentile of each experiment with a line marking the median. Whiskers extend to the minimum and maximum

*CrDELLA*-silenced leaves displayed a significant increase in leaf length: width ratios (Fig. 4 and 43% increase in immature leaves, *p* = 0.0004; 30% increase in mature leaves, *p* = 0.04). This phenotype was previously observed in *C. roseus* plants exposed to GA, which would lead to DELLA degradation (Srivastava and Srivastava 2007). In contrast, *CrGID1*-silenced plants displayed a consistent crinkling of leaves (Fig. 3B and C, Figure S4), similar to previous observations of *GID1* knockout mutants in tomato plants (Illouz-Eliaz et al. 2019). These changes in leaf shape with *CrDELLA*-silencing and *CrGID1*-silencing were seen in both silenced leaf pairs but were strongest in the immature leaf pair (Figs. 3B and C and 4).

In addition to changes in leaf shape, leaves of *CrDELLA*-silenced plants appeared to bend upward (hyponasty) while leaves in *CrGID1*-silenced plants were horizontally aligned (epinasty). Hyponasty and elongation of stems and leaves are phenotypes of the shade avoidance syndrome (SAS) (Casal 2012) and have been previously observed in other *DELLA* knockout plants (Djakovic-Petrovic et al. 2007; Küpers et al. 2023). This constitutive SAS response of DELLA-silenced plants is likely caused by DELLAs’ interactions with light-signaling factors like PIFs (Küpers et al. 2023) (Figure S2); silencing DELLAs removes their inhibition of PIFs, which can then activate SAS.

Overall, we observed growth-related phenotypes in *CrDELLA*-silenced and *CrGID1*-silenced *C. roseus* plants opposite to each other and consistent with literature. These visual phenotypes supported our identification of CrDELLAs and CrGID1s and confirmed that partial and transient silencing with VIGS was sufficient to yield visible changes.

### Influence of *CrDELLA* or *CrGID1* silencing on vindoline pathway gene expression

We next investigated the effect of *CrDELLA* and *CrGID1* silencing on the expression of the vindoline pathway genes and on terpenoid indole alkaloid accumulation (next section). Since GID1 degrades DELLAs, viral induced gene silencing of *CrGID1a* and *CrGID1b* served as a complementary method of confirming CrDELLA function. Silencing *CrDELLA* genes would be expected to decrease DELLA protein levels while silencing *CrGID1* genes would be expected to increase DELLA protein levels. RNA was extracted from either the first or second leaf pair to emerge after infection (mature or immature leaves, respectively), and gene expression was monitored with qPCR.

In the first experiment, we observed 60–70% silencing of *CrDELLA1* and *CrDELLA2* in immature leaves (Fig. 5A, *p* < 0.0001), 50–60% silencing of *CrDELLA1* and *CrDELLA2* in mature leaves (Fig. 5B, *p* < 0.001), 75% silencing of *CrGID1a* (*p* < 0.001) and 30% silencing of *CrGID1b* (*p* = 0.3) in immature leaves (Fig. 5C), and 45% silencing of *CrGID1a* (*p* = 0.07) and 50% silencing of *CrGID1b* in mature leaves (*p* = 0.1) (Fig. 5D). *CrGID1b* already has low basal expression in leaves, which may explain why it was not strongly silenced (Figure S1).

In the first experiment, there was moderate evidence that silencing *CrDELLA1* and *CrDELLA2* decreased vindoline pathway gene expression in immature leaves, suggesting that DELLAs positively regulate vindoline biosynthesis. There were significant 20–30% decreases in the expression of *T3O*, *T3R*, *NMT*, and *D4H* with *CrDELLA*-silencing (*p* = 0.03, 0.01, 0.02, 0.008, respectively) (Fig. 5A). In mature leaves, only *D4H* expression significantly decreased with *CrDELLA*-silencing (45% decrease, *p* = 0.02) (Fig. 5B). In contrast, upstream TIA pathway genes, *TDC* and *G10H*, did not show any significant changes with *CrDELLA*-silencing in either immature or mature leaves (Figure S5), suggesting that CrDELLAs might specifically regulate the downstream vindoline pathway.

In the first experiment, there was weak evidence that silencing *CrGID1a* and *CrGID1b*, which should increase DELLA stability, increased vindoline pathway gene expression in mature leaves. Many of the same genes that decreased with *CrDELLA*-silencing in immature leaves increased with *CrGID1*-silencing in mature leaves: there were 1.3–2.8-fold increases in expression of *T3O*, *NMT*, *D4H*, and *DAT* with *CrGID1*-silencing (*p* = 0.2 for each gene) (Fig. 5D). In immature leaves, there was no effect on vindoline pathway gene expression with *CrGID1*-silencing (Fig. 5C, Figure S6). This first experiment supported the hypothesis that CrDELLAs could activate expression of the vindoline pathway.

We repeated these experiments two more times, for a total of 3 experimental repeats. Due to observed decreases in vindoline pathway gene expression with *CrDELLA*-silencing in immature leaves and increases in vindoline pathway gene expression with *CrGID1*-silencing in mature leaves, we only measured gene expression in these leaves in the subsequent two experiments (i.e. immature leaves for *CrDELLA*-silencing, and mature leaves for *CrGID1*-silencing).

Across the three experiments, we observed 60–70% silencing of *CrDELLA1* and *CrDELLA2* in immature leaves (Fig. 6A, *p* < 0.0001) and about 50% silencing of *CrGID1a* and *CrGID1b* in mature leaves (Fig. 6B, *p* = 0.0007 and 0.03 for *CrGID1a*- and *CrGID1b*-silencing, respectively). In contrast to the first experiment, the following two experiments showed little to no effect on vindoline pathway gene expression with *CrDELLA*-silencing or *CrGID1*-silencing. On average, *NMT*, *D4H*, and *DAT* expression decreased by 20% with *CrDELLA*-silencing, and increased by 20–30% with *CrGID1*-silencing, but these changes were not statistically significant (*p* = 0.2–0.6). As detailed in the Discussion, variability in silencing efficiency, leaf developmental state (Fig. 4A), or environmental conditions between experiments likely contributed to the difficulty in replicating results and establishing strong evidence. Overall, silencing *CrDELLA* or *CrGID1* provided only weak evidence to support our hypothesis that CrDELLAs positively regulate vindoline pathway gene expression.

### Influence of *CrDELLA* or *CrGID1* silencing on terpenoid indole alkaloid accumulation

Next, we explored whether silencing *CrDELLA* or *CrGID1* affected TIA metabolite levels. Alkaloids were extracted from leaves of VIGS plants and the levels of four key TIAs (vindoline, catharanthine, ajmalicine, and serpentine) were monitored using HPLC-MS-MS. We additionally looked for tabersonine, vinblastine, and vincristine, but levels were too low to detect.

We observed no changes in TIA levels in *CrDELLA*-silenced immature leaves (Fig. 7A, Figure S7) or *CrGID1*-silenced mature leaves (Fig. 7B). However, there was strong evidence for *CrGID1*-silencing increasing TIA levels in immature leaves (Fig. 7C). In the first experiment, there was a 1.9-fold increase in vindoline (*p* < 0.0001), a 1.6-fold increase in catharanthine (*p* = 0.006), a 2.8-fold increase in ajmalicine (*p* = 0.01), and a 1.5-fold increase in serpentine (*p* = 0.04). Silencing *CrGID1* should lead to increased CrDELLA protein stability, so these increases in TIA levels with *CrGID1*-silencing support our hypothesis that CrDELLAs can positively regulate vindoline biosynthesis and suggest that CrDELLAs might activate TIA biosynthesis in general.

Due to these significant increases in immature leaves of *CrGID1*-silenced plants, we repeated TIA analysis in immature leaves in a second experiment. In this second experiment, these increases were not repeated and there was no evidence that *CrGID1*-silencing impacted TIA levels (Fig. 7D). The efficiency of *CrGID1a* silencing in this second experiment was much lower than the first experiment – about 75% silencing of *CrGID1a* in the first experiment compared to 50% silencing of *CrGID1a* in the second experiment (Fig. 7E). This lower silencing efficiency could potentially explain why we did not observe increases in TIA levels in the second experiment.

Similar to gene expression results, the first silencing experiment exhibited strong evidence that silencing *CrGID1* could increase TIA biosynthesis, but these results were not reproduced in a second experiment. As detailed in the Discussion, variability in silencing efficiency, leaf developmental state, or environmental conditions likely contributed to the difficulty in replicating results and establishing strong evidence. Overall, silencing *CrDELLA* or *CrGID1* provided weak evidence supporting our hypothesis that CrDELLAs can activate vindoline biosynthesis. However, DELLAs are strongly regulated post-translationally (Blanco-Touriñán, Serrano-Mislata, et al., 2020b), and so transcript levels might not accurately reflect protein levels. We next explored potential methods for increasing DELLA protein levels in an attempt to increase vindoline biosynthesis and further explore the hypothesis that CrDELLAs activate vindoline biosynthesis.

### Application of paclobutrazol, a gibberellic acid inhibitor, to etiolated *C. roseus* seedlings

**Fig. 8:**
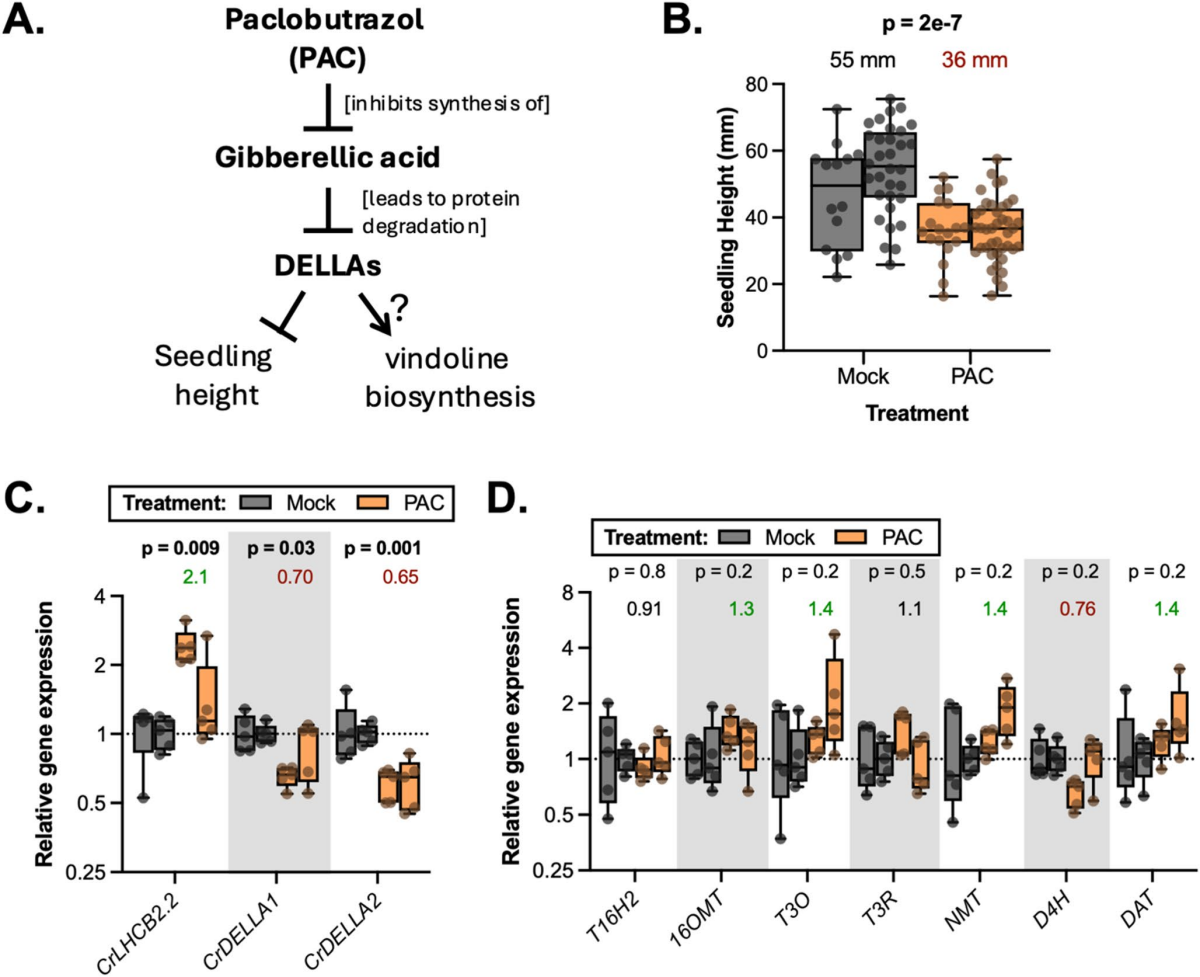
There is weak evidence that treating etiolated seedlings with paclobutrazol (PAC), an inhibitor of GA synthesis, increases vindoline pathway gene expression. (**A**) PAC mechanism: PAC inhibits the biosynthesis of GA (Rademacher 2000). GA leads to the degradation of DELLAs (Davière and Achard 2013), so application of PAC leads to an increase in DELLA protein content in the dark when GA levels are normally high (Djakovic-Petrovic et al. 2007; Oh et al. 2007). DELLAs inhibit plant height (Peng et al. 1999); in this study, we are investigating the role DELLA may play in regulating vindoline biosynthesis. (**B**) Application of 1 µM PAC to etiolated seedlings decreased seedling height compared to the mock treatment (DMSO). Height was measured using ImageJ. Each replicate is an individual seedling (*n* = 14–41 per experiment), and the experiment was repeated twice (displayed as separate but adjacent boxes). Significance of the effect of PAC treatment is indicated with stars (**** *p* < 0.0001) according to a two-way ANOVA analyzing a full-factorial model for variables “gene-silenced” and “experimental repeat”. (**C**) PAC treatment increased *CrLHCB2.2* gene expression (positive control) and decreased *CrDELLA1* and *CrDELLA2* expression. (**D**) There was weak evidence that PAC treatment increased expression of some vindoline pathway genes (*T3O*,* NMT*, and *DAT*). For gene expression, each biological replicate is a pool of 3 whole seedlings (*n* = 5). The experiment was repeated twice (displayed as separate but adjacent boxes). Relative gene expression was measured with qPCR and calculated using the 2^−∆∆Ct^ method (Livak and Schmittgen 2001) relative to the control condition (mock treatment), and normalized relative to the housekeeping gene, SAND (Pollier et al. 2014). Numbers above the boxes represent the median for all of the experiments combined (> 1.2 is in green, < 0.85 is in red). P-values indicate significance of the effect of PAC treatment according to a two-way ANOVA on ∆∆Ct values, using a full-factorial model for variables “PAC treatment” and “experimental repeat”. P-values were corrected for false discovery rate (FDR = 5%). P-values less than 0.05 are bolded. Complete ANOVA results can be found in Supplemental data 1. Boxes represent the 25th and 75th percentile with a line marking the median. Whiskers extend to the minimum and maximum

To further determine whether increasing CrDELLA protein levels could increase vindoline biosynthesis, we treated etiolated (never exposed to light) *C. roseus* seedlings with paclobutrazol (PAC), a chemical that inhibits GA biosynthesis (Rademacher 2000). In the dark, GA levels are high and DELLA proteins are degraded (Kamiya and García-Martínez 1999; Reid et al. 2002; Xu et al. 2021; Zhong et al. 2021). PAC inhibits GA synthesis and thus increases DELLA protein levels in the dark (Djakovic-Petrovic et al. 2007; Oh et al. 2007) (Fig. 8A). We performed this experiment twice.

Due to GA’s positive role in growth, PAC treatment would be expected to inhibit seedling growth. Consistent with this mechanism, we observed a significant 30% decrease in seedling height in PAC-treated seedlings in both experiments (Fig. 8B, *p* < 0.0001). As a positive control, we measured expression of a homolog of the light harvesting complex subunit B 2.2, *CrLHCB2.2*, which was previously shown to be activated by PAC treatment of dark-grown *A. thaliana* seedlings (Cheminant et al. 2011). PAC treatment increased *CrLHCB2.2* gene expression by about 2-fold (Fig. 8C, *p* = 0.009), further confirming that PAC treatment was impacting seedling physiology as expected.

There was weak evidence that PAC (and thus expected higher CrDELLA levels) increased vindoline pathway gene expression. Across both experiments, we observed 40% increases in *T3O*, *NMT*, and *DAT* gene expression (*p* = 0.2) (Fig. 8D). Increases in vindoline pathway gene expression with PAC treatment are consistent with previously observed increases in vindoline content (15%) in roots of PAC-treated *C. roseus* plants (Jaleel et al. 2009), and provides additional evidence for DELLAs positively regulating the vindoline pathway.

Although DELLA protein levels are expected to increase with PAC treatment (Djakovic-Petrovic et al. 2007; Oh et al. 2007), we observed a 30–35% decrease in *CrDELLA1* and *CrDELLA2* transcript levels (*p* = 0.03, and 0.001, respectively) (Fig. 8C). This likely indicated activation of negative feedback loops. For example, DELLAs can inhibit PIF1/PIL5, which directly activate DELLA expression (Li et al. 2016; Oh et al. 2007); therefore, the stabilization of DELLAs with PAC would be expected to decrease *CrDELLA* expression (negative feedback), reducing the effects of PAC treatment on vindoline pathway gene expression and potentially explaining the 24% decrease in *D4H* expression (*p* = 0.2).

Overall, PAC treatment provided weak evidence supporting our hypothesis that CrDELLAs can activate expression of some vindoline pathway genes (*T3O*, *NMT*, and *DAT*).

### Overexpression of N-terminal truncated *CrDELLA1* and *CrDELLA2* in *C. roseus* seedlings

As complementary evidence to the VIGS experiments, we investigated the effect of overexpressing the N-terminal truncated CrDELLA on regulation of the vindoline pathway. We saw no effect on vindoline pathway gene expression when full-length *CrDELLA1* was overexpressed in *C. roseus* seedlings (Figure S8), likely due to rapid degradation of the full-length DELLA protein. Truncating the N-terminus of DELLAs inhibits their interaction with GID1 and leads to their stabilization (Boss and Thomas 2002; Cassani et al. 2009; Harberd and Freeling 1989; Hirano et al. 2010; Muangprom et al. 2005; Peng et al. 1997, 1999). We thus cloned *CrDELLA1∆1-112* and *CrDELLA2∆1-113* (amino acids 1-112 or 1-113 removed), which truncated the full DELLA domain and part of the TVHYNP domain, mimicking similar truncations that led to stabilization and gain-of-function phenotypes of DELLA proteins in wheat (Peng et al. 1999). Truncated *CrDELLA*s were overexpressed in *C. roseus* seedlings and vindoline pathway promoter activity was monitored using a dual luciferase assay. Beta-glucuronidase (*GUS*) was overexpressed as a negative control.

**Fig. 9:**
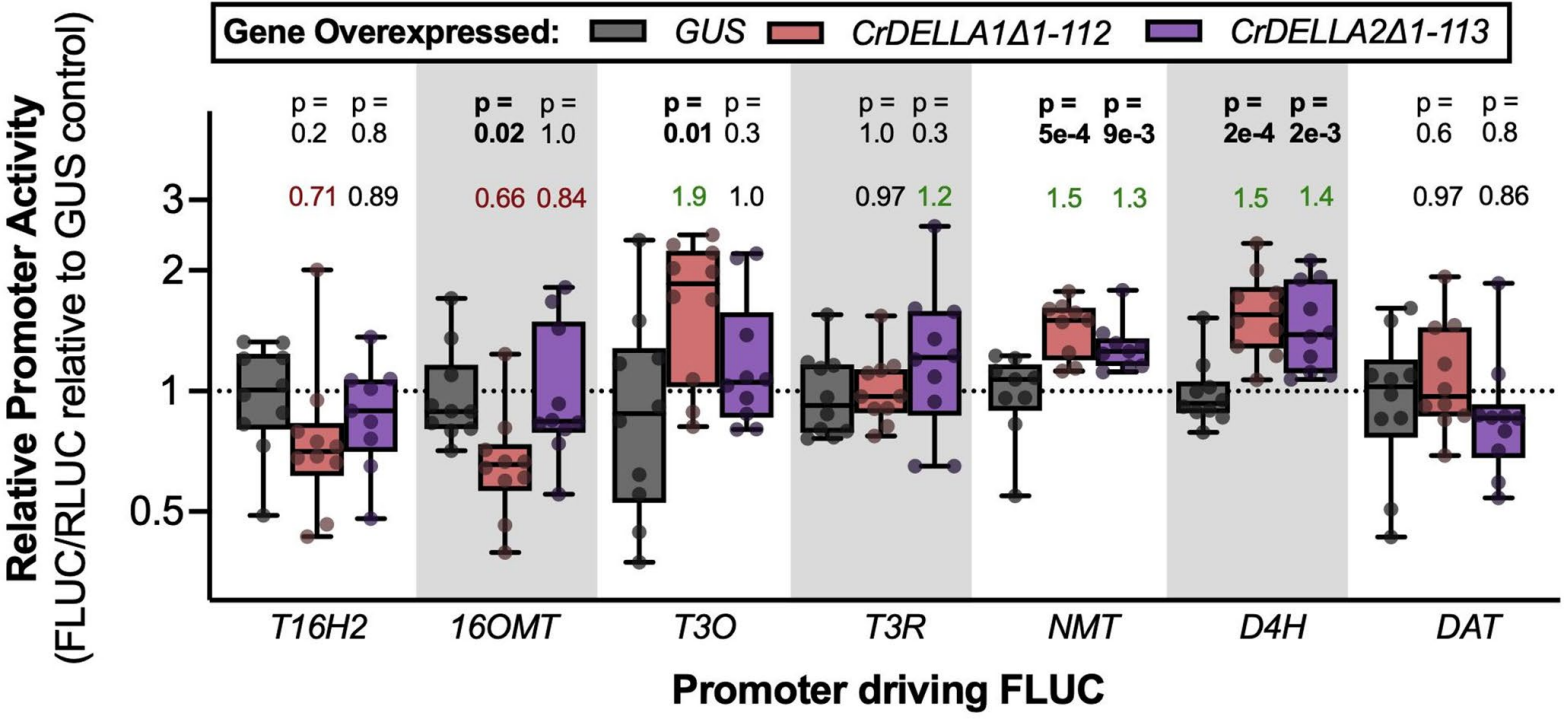
Overexpression of truncated *CrDELLA*s activated some vindoline pathway promoters. *C. roseus* seedlings were transformed with two strains of *A. tumefaciens* in a 1:1 ratio: (**I**) strain containing an effector plasmid, consisting of the *Arabidopsis thaliana Ubiquitin 10* (*AtUbi10*) promoter driving the expression of the truncated *CrDELLA1∆1-112*, *CrDELLA2∆1-113*, or intron-containing *GUS* (negative control) (**II**) strain containing a vindoline pathway promoter driving the intron-containing firefly luciferase (*FLUC)* and the *AtuNOS* promoter driving intron-containing *Renilla* luciferase (*RLUC)* as an internal control. CrDELLA1 and CrDELLA2 were truncated at the N-terminus to prevent protein degradation. The relative promoter activity is the ratio of FLUC to RLUC luminescence for each sample normalized to the average FLUC to RLUC ratio of the *GUS*-overexpressed negative control. Each biological replicate is a pool of 2 seedlings (*n* = 10). Numbers above the boxes represent the median promoter activity, relative to *GUS*-overexpressed (> 1.2 is in green, < 0.85 is in red). P-values were calculated from a one-way ANOVA (adjusted for FDR = 5%) followed by Dunnett’s post-hoc test. P-values less than 0.05 are bolded. Boxes represent the 25th and 75th percentile with a line marking the median. Whiskers extend to the minimum and maximum

There was moderate to strong evidence that overexpressing *CrDELLA1∆1-112* or *CrDELLA2∆1-113* increased *NMT* and *D4H* promoter activity by 30–50% (*p* < 0.01) (Fig. 9). Overexpressing *CrDELLA1∆1-112* also increased *T3O* promoter activity (90% increase, *p* = 0.01) and decreased *16OMT* promoter activity (34% decrease, *p* = 0.02). Overall, overexpressing truncated *CrDELLAs* provided moderate evidence that both CrDELLA1 and CrDELLA2 can activate some vindoline pathway promoters (*T3O*, *NMT*, and *D4H*); the activation of the vindoline pathway promoters with truncated *CrDELLAs* is also evidence that increasing or stabilizing CrDELLA protein levels, rather than just increasing native *CrDELLAs* transcript levels, is necessary for increasing the expression of the vindoline pathway.

## Discussion

We identified two DELLA and two GID1 proteins in the important medicinal plant *Catharanthus roseus*, confirmed their role in regulating growth and development, and functionally characterized their role in regulating the vindoline biosynthesis pathway. DELLAs are integrators of many signaling pathways; they positively influence light signaling (Achard et al. 2007; Blanco-Touriñán et al. 2020a; de Lucas et al. 2008; Djakovic-Petrovic et al. 2007; Feng et al. 2008; Gallego-Bartolomé et al. 2010; Lee et al. 2022; Li et al. 2016; Xu et al. 2021; Zhong et al. 2021), JA-mediated defense signaling (Hou et al. 2010; Leone et al. 2014; Wild et al. 2012; Xie et al. 2016; Yang et al. 2012), and maintenance of a juvenile leaf state (Chen et al. 2014, 2017; Lei et al. 2020; Yu et al. 2012; Zhang et al. 2018, 2021). Similarly, the vindoline pathway is highly expressed in young leaves, activated by light, and activated by JA in a light and developmentally dependent manner (Aerts et al. 1994; Besseau et al. 2013; Cole-Osborn et al. 2024a; Góngora-Castillo et al. 2012; Hernández-Domínguez et al. 2004; Liscombe et al. 2010a; Liu et al. 2019; Mall et al. 2019; Qu et al. 2015; Raina et al. 2012; Schröder et al. 1999; St-Pierre et al. 1998, 1999; van der Fits & Memelink, 2000b; Vázquez-Flota and De Luca 1998; Vazquez-Flota and De Luca 1998; Wang et al. 2010; Wei 2010; Yu et al. 2018b; Zhou et al. 2015). We thus hypothesized that CrDELLAs might activate vindoline biosynthesis.

To explore this hypothesis, we perturbed gene expression and protein levels of *CrDELLA*s and *CrGID1*s in *C. roseus* using different approaches (i.e. VIGS, inhibition of GA biosynthesis, overexpression); we then measured the effects of these perturbations on multiple layers of vindoline pathway regulation, either promoter activity, gene expression, or alkaloid levels. Each of these layers involves different regulatory mechanisms and kinetics. We first explain how this affects the interpretation of the results before summarizing our findings from the different experimental approaches for evaluating the role of *CrDELLA* and *CrGID1*. For example, after JA addition to *C. roseus* hairy root cultures, maximum expression of TIA genes occurred between 8 and 24 h followed by the maximum metabolite accumulation after 3–5 days (Goklany et al. 2013; Rizvi et al. 2016). The kinetics of the gene expression and alkaloid production layers is particularly important in interpreting the VIGS findings. VIGS of *C. roseus* plants requires 2–3 weeks for maximum silencing of the target gene. When sampling during this period, it is unclear which snapshot is captured during this dynamic regulation of gene expression and alkaloid production. This may explain why changes in vindoline pathway gene expression levels did not always correlate with changes in vindoline and other alkaloid levels. For example, in the first *CrDELLA*-silencing experiment, we observed significant decreases in vindoline pathway gene expression but no change in vindoline and other alkaloid levels in young leaves. In contrast, in the first *CrGID1*-silencing experiment, we observed no changes in vindoline pathway gene expression but saw significant increases in vindoline and other alkaloid levels in young leaves. We may have caught the appropriate window for gene expression changes but not metabolite accumulation in the first *CrDELLA*-silencing experiment. Similarly, we may not have caught the appropriate window for gene expression changes but did for metabolite accumulation in the first *CrGID*-silencing experiment. Thus, due to their different regulatory mechanisms and kinetics, either increased promoter activity, gene expression, or alkaloid levels is positive evidence supporting our hypothesis that CrDELLAs regulate vindoline biosynthesis.

Overall, transiently silencing *CrDELLA1* and *CrDELLA2* or *CrGID1a* and *CrGID1b*, treating *C. roseus* seedlings with the GA-synthesis inhibitor PAC, or transiently overexpressing truncated *CrDELLA1∆1-112* or *CrDELLA2∆1-113* provided weak to moderate evidence that CrDELLAs can act as activators of vindoline biosynthesis, particularly for *T3O*,* NMT*, *D4H*, and *DAT*. The strength of this evidence is likely limited by the complexity of the GA signaling pathway (negative feedback illustrated in Fig. 8) and limitations of controlling this system with only transient expression methods and controlling plant growth in ambient environmental conditions. Despite these limitations, this initial investigation into the activity of CrDELLAs and CrGID1s in *C. roseus* suggests that manipulation of this signaling pathway in transgenic plants has potential to increase production of the precursors to the important chemotherapies, vinblastine and vincristine.

When we first silenced *CrDELLA1* and *CrDELLA2* or *CrGID1a* and *CrGID1b* in *C. roseus* leaves, we observed 20–30% significant decreases in *T3O*, *T3R*, *NMT*, and *D4H* expression with *CrDELLA*-silencing in immature leaves (*p* = 0.008–0.03) (Fig. 5A) and 1.3–2.8-fold increases in *T3O*, *NMT*, *D4H*, and *DAT* expression with *CrGID1*-silencing in mature leaves (*p* = 0.2) (Fig. 5D). When this experiment was repeated two more times, we did not observe significant changes in vindoline pathway gene expression with *CrDELLA*- or *CrGID1*-silencing, but on average, we still observed 20% decreases in *NMT*, *D4H*, and *DAT* expression with *CrDELLA*-silencing in immature leaves (*p* = 0.2–0.6) (Fig. 6A) and 20–30% increases in *NMT*, *D4H*, and *DAT* expression with *CrGID1*-silencing in mature leaves (*p* = 0.3–0.4) (Fig. 6B). Similarly, we first observed 1.5–2.8-fold significant increases in vindoline, catharanthine, ajmalicine, and serpentine levels with *CrGID1*-silencing in immature leaves (*p* < 0.0001 for vindoline) (Fig. 7C). However, no changes in TIA accumulation were observed in a second experiment (Fig. 7D). PAC application on etiolated seedlings (presumably increasing DELLA stability) led to 1.4-fold increases in *T3O*, *NMT*, and *DAT* expression (*p* = 0.2) (Fig. 8D). Finally, overexpression of the N-terminal truncated and stabilized *CrDELLA1∆1-112* or *CrDELLA2∆1-113* significantly increased *NMT* and *D4H* promoter activity by 30–50% (*p* < 0.01) (Fig. 9). DELLAs are subject to post-translational regulation (Blanco-Touriñán, Serrano-Mislata, et al., 2020b); thus altering transcript levels alone with overexpressing the native *CrDELLAs* may not be sufficient to alter its protein levels; instead, overexpressing the truncated *CrDELLAs* promoted stable and increased protein levels. Taken together, these three experimental approaches provided weak to moderate evidence for a positive role of CrDELLAs in regulating vindoline biosynthesis. We discuss reasons for the variability between silencing experiments and propose approaches for addressing and strengthening the evidence of CrDELLAs’ positive role below.

One reason for the low replicability between independent silencing experiments is due to the complexity of the GA signaling pathway. The GA signaling pathway contains multiple negative feedback loops that help maintain homeostasis in the native system but hinders perturbations during experimental study. For example, DELLAs activate transcription of *GID1* (Cao et al. 2006; Griffiths et al. 2006), activate biosynthesis of active GAs (Fukazawa et al. 2017; Griffiths et al. 2006; Illouz-Eliaz et al. 2019), and inhibit activators of their own expression, like PIF1/PIL5 (Li et al. 2016; Oh et al. 2007). This negative feedback was evident in PAC-treated *C. roseus* seedlings, where we observed a significant decrease in *CrDELLA* transcript levels under conditions that presumably increase DELLA protein stability (Fig. 8C). Partial silencing of *CrDELLA* and *CrGID1* may not have sufficiently perturbed this system that tends towards homeostasis. In addition, DELLAs serve as both positive and negative regulators of JA signaling, depending on timing and context. DELLAs can inhibit JAZ, releasing MYC activators, but DELLAs can also bind and inhibit MYCs (Frerigmann et al. 2021; Hong et al. 2012).

Another reason for the low replicability between independent silencing experiments is due to the variable and limited extent of silencing with only transient expression methods. Methods for constructing fully transgenic *C. roseus* plants are limited, time-consuming and inefficient (Bomzan et al. 2022; Choi et al. 2004; Kumar et al. 2018; Pan et al. 2012; Sharma et al. 2018; Verma et al. 2022; Verma and Mathur 2011; Wang et al. 2012). For this reason, we began our characterization of *CrDELLA* and *CrGID1* genes in *C. roseus* using more rapid transient gene silencing and transient gene overexpression. Variability in our VIGS results may have been partially caused by incomplete silencing. For example, in the first experiment, we observed 75% silencing of *CrGID1a* and significant increases in TIA levels. In contrast, in the second experiment, we observed only 50% silencing of *CrGID1a* and no changes in TIA levels (Fig. 7E). Future experiments could better control this complex system and reduce variability by completely knocking out *CrDELLAs* or *CrGID1s* in *C. roseus* plants or tissue cultures.

Another source of variability in these silencing experiments were environmental differences in plants grown under ambient temperature and humidity. GA levels are strongly linked to development and are influenced by environmental factors like temperature and drought stress (Colebrook et al. 2014; Yamaguchi 2008). Disruption of normal GA-signaling through partial silencing of *DELLA*s and *GID1*s can increase phenotypic variability in response to these changing environmental conditions. For example, Illouz-Eliaz et al. previously reported that partial *GID1* mutants exhibited phenotypic instability under ambient, non-optimal environments; under greenhouse conditions, plant weight coefficients of variation (CVs) were 48–105% when 2 out of 3 *GID1* genes were knocked out as compared to 26% for wild type tomato plants (Illouz-Eliaz et al. 2019). In the future, growing plants in an environmentally controlled growth chamber might reduce variability within and between experiments and strengthen experimental evidence.

Despite the variability that we observed, our results indicate that under certain conditions, silencing *CrGID1* could increase vindoline and catharanthine levels by 60–90% (Fig. 7C). If we can identify and control the factors that caused inconsistent results with *CrGID1* silencing, this could lead to significant boosts in production of vindoline and catharanthine, the two immediate precursors to the chemotherapeutics vinblastine and vincristine. Literature also suggests that DELLAs are promising and practical targets for genetic engineering. Gain-of-function (GOF) DELLA mutants are healthy semi-dwarf plants widely used in agriculture (Hedden 2003). Natural dwarf varieties of *C. roseus* suggest that GOF mutation of CrDELLAs may be a promising route to engineering increased production of vinblastine and vincristine. Consistently, *C. roseus* cultivars or mutants identified as dwarf and semi-dwarf varieties produce the highest levels of TIAs (Heijden et al. 2005; Kulkarni et al. 1999; Mall et al. 2021). It is possible that CrDELLAs contribute to these high TIA levels in dwarf *C. roseus* plants.

This study is the first identification and characterization of *DELLA* and *GID1* genes in *C. roseus*. Using transient expression methods, we provide weak to moderate evidence supporting the role of CrDELLAs in positively regulating vindoline biosynthesis. Future development of transgenic *C. roseus* plants with modified *CrDELLA* or *CrGID1* expression could lead to healthy mutant plants with increased production of critical chemotherapy medicines.

## Electronic supplementary material

Below is the link to the electronic supplementary material.

Supplementary Material 1

## Data Availability

Vindoline pathway reporter plasmids were deposited at Addgene (IDs: 203896–203902). Datasets generated during the current study are available from the corresponding author on reasonable request.
