# Supplementary material for: Identification of DELLA and GID1 genes in Catharanthus roseus and their potential role in regulating vindoline biosynthesis
Source: Plant Mol Biol. 2025 Jun 5;115(3):72. doi: 10.1007/s11103-025-01599-1 (PMC12141139; doi:10.1007/s11103-025-01599-1)
Supplement: Supplementary file 1 — Supplementary Material 1 [file 11103_2025_1599_MOESM1_ESM.docx]

**Identification of *DELLA* and *GID1* genes in *Catharanthus roseus* and their role in regulating vindoline biosynthesis**

Lauren F. Cole-Osborn^1,2^, Natalie Soens^1^, Diana Bernal-Franco^1,3^, Olga Prifti^2^, Erin J. Cram^3^, Carolyn W.T. Lee-Parsons^1,2,4,5^

^1^ Northeastern University, Department of Chemical Engineering, Boston, MA, USA 02115

^2^ Northeastern University, Department of Bioengineering

^3^ Northeastern University, Department of Biology

^4^ Northeastern University, Department of Chemistry and Chemical Biology

^5^ Corresponding author. ca.lee@northeastern.edu. ORCID = 0000-0001-5905-1214

## Supplementary Figures and Tables

Table S1. Primers used in this study.

Uppercase indicates sequences complementary to its target while lowercase indicates 5’ overhangs to facilitate cloning.

| **Primer Name** | **Primer Sequence** | **Purpose** | **Source** |
| --- | --- | --- | --- |
| DELLAFattb1_2 | ggggacaagtttgtacaaaaaagcaggctggATGAAGAGGGACCATACG | Amplification of *CrDELLA1* and *CrDELLA2* for Y2H Assay  (Gateway compatible) | This study |
| DELLARattb2 | ggggaccactttgtacaagaaagctgggtagtTTAACCGAGTTTCCAG |  |  |
| RH_DELLA_trunc1_F | ATGGGCGTTTTTGGGATTCCAAATGAGTCTG | N-terminal truncation of *CrDELLA1* for Y2H (via round-the-horn PCR) | This study |
| RH_DELLA_trunc1_R | CCAGCCTGCTTTTTTGTACAAAGTTGG |  |  |
| PIF4Fattb1_2 | ggggacaagtttgtacaaaaaagcaggctctATGTATCCTTGCTTTCCTG | Amplification of *CrPIF4/5* for Y2H Assay  (Gateway compatible) | This study |
| PIF4Rattb2 | ggggaccactttgtacaagaaagctgggtactTCAGCCTAGACTTTGC |  |  |
| JAZ1Fattb1_2 | ggggacaagtttgtacaaaaaagcaggctcgATGGATAAATCTGGCCAG | Amplification of *CrJAZ1∆1-84* for Y2H Assay  (Gateway compatible) | This study |
| JAZ1Rattb2 | ggggaccactttgtacaagaaagctgggtgggTTTTAAAAAGGAAAGCC |  |  |
| LC19_DELLA1_F | aagaagacaaaATGAAGAGGGACCATACGAAAAC | Amplification of *CrDELLA1* for overexpression  (MoClo compatible) | This study |
| LC20_DELLA1_R | aagaagacaaaagcTTAACCGAGTTTCCAGGCCG |  |  |
| LN03_ Fwd_Della1and2_trunc | ttgaagacaaaATGATTTCTGAAATCAACCCTGATG | Amplification of *CrDELLA1∆1-112* and *CrDELLA2∆1-113* for overexpression  (MoClo compatible) | This study |
| LN04_ Rev_Della1_trunc | ttgaagacaaaagcTTAACCGAGTTTCCAGGCC |  |  |
| LN05_ Rev_Della2_trunc | ttgaagacaaaagcTTAACCGAGTTTCCAGGCTG |  |  |
| LC59_DELLA1_V1_F | tttggtctcatatgACCCTTTTCGATTCACTTG | Amplification of *CrDELLA1* fragment for VIGS silencing | This study |
| LC60_DELLA1_V1_R | tttggtctcaatttCACTCTATAGCCATCTCCAC |  |  |
| LC61_DELLA2_V1_F | tttggtctctAAATTCGCTCACTTTACAGC | Amplification of *CrDELLA2* fragment for VIGS silencing | This study |
| LC62_DELLA2_V1_R | tttggtctcacaccTGCATCAAGATCAGCAAG |  |  |
| LC82_GID1a_VIGS_F | tttggtctcatatgCTATTTGTATGATATCAAGATTCG | Amplification of *CrGID1a* fragment for VIGS silencing | This study |
| LC83_GID1a_VIGS_R | tttggtctcaggcaATGCCAACTTGAAATTTGAG |  |  |
| LC84_GID1b_VIGS_F | tttggtctctTGCCGTAGATTAGTTAACA | Amplification of *CrGID1b* fragment for VIGS silencing | This study |
| LC85_GID1b_VIGS_R | tttggtctcacaccTGATTCTGTTCTTGTTTCTC |  |  |
| DELLA1_qF | GCTGAGGCTATCCAACAAGAA | qPCR amplifying *CrDELLA1* | This study |
| DELLA1_qR | GCCAATGCTTCAGCGAAATAG |  |  |
| DELLA2_qF | GGTATGGCAAAGATGTGGAAAG | qPCR amplifying *CrDELLA2* | This study |
| DELLA2_qR | CAGCCATGTCCGAGGATTTA |  |  |
| GID1a_qF | TTGCTGGGCTGCTGTAAA | qPCR amplifying *CrGID1a* | This study |
| GID1a_qR | CCACCAGAACTATCACCACATAA |  |  |
| GID1b_qF | CTAACTTCAAGCTGGCCTACA | qPCR amplifying *CrGID1b* | This study |
| GID1b_qR | GAATTGTGTTAGCAGGGACTTTC |  |  |
| q2T16h_up | GATCAACTCACAGTGGCAGTC | qPCR amplifying *T16H2* | (L. F. Cole-Osborn et al., 2024) |
| q2T16h_down | GACTTGAGACTTGTGATTGGC |  |  |
| 16OMTvigs-qF1 | GTGTGAAGATACTCAAAAGCTGC | qPCR amplifying *16OMT* | (Liscombe & O’Connor, 2011) |
| 16OMTvigs-qR1 | CAAAATTTACAAGCATTGCCATATCC |  |  |
| T3O_qF | GTCATAGACGAGCACAGAGAAA | qPCR amplifying *T3O* | (L. F. Cole-Osborn et al., 2024) |
| T3O_qR | CACCACCCTCTTCAATCCTAAG |  |  |
| T3R_qF | CTTGAGCCACTCTTTGCTTTAC | qPCR amplifying *T3R* | (L. F. Cole-Osborn et al., 2024) |
| T3R_qR | ATGAGGGACATTGCGGATAC |  |  |
| 2270-qF1 | TGACAAAGTAACCGGAGCATGGGA | qPCR amplifying *NMT* | (Liscombe & O’Connor, 2011) |
| 2270-qR1 | ATCCGAATGACGGCATCTTGGCTA |  |  |
| D4Hvigs-QF1 | TGGCCTCAGTAGCAATTCAG | qPCR amplifying *D4H* | (Liscombe & O’Connor, 2011) |
| D4Hvigs-QR1 | TCCATATTTCTCACTCGCTTCTC |  |  |
| DATvigs-qF1 | GAGGTTTTGACTGCTTTTCTCAG | qPCR amplifying *DAT* | (Liscombe & O’Connor, 2011) |
| DATvigs-qR1 | TGGAAATGGCAAAGATTGGC |  |  |
| LHCB2_qF | GTTGTTCTCATGGGCTTGATTG | qPCR amplifying *CrLHCB2.2* | (L. F. Cole-Osborn et al., 2024) |
| LHCB2_qR | AATGCTCCTCCTGGGTAGAT |  |  |
| SAND_qF | TGCTGTGGAGGAGGAAGAAG | qPCR amplifying *SAND* | (Pollier, Vanden Bossche, Rischer, & Goossens, 2014) |
| SAND_qR | ACTGGCGGAACTACTACTACC |  |  |


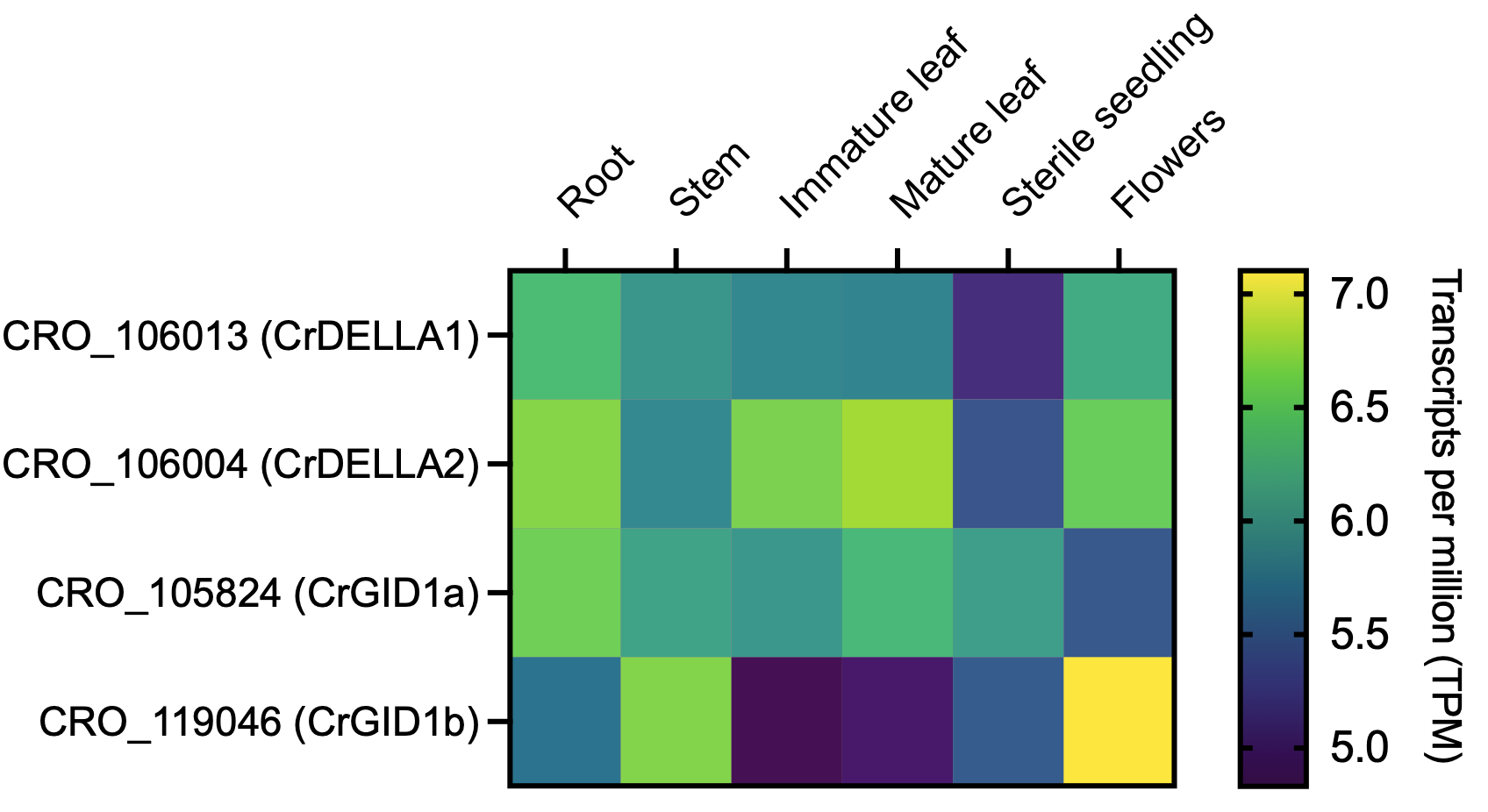


**Figure S1.** **Tissue-specific expression levels of *CrDELLA* and *CrGID1* genes.** Transcripts per million (TPM) values were calculated from previously published RNAseq data (Góngora-Castillo et al., 2012) mapped to the *C. roseus* v. 2 genome (Franke et al., 2019), downloaded from KBase (L. Cole-Osborn et al., 2022).

**
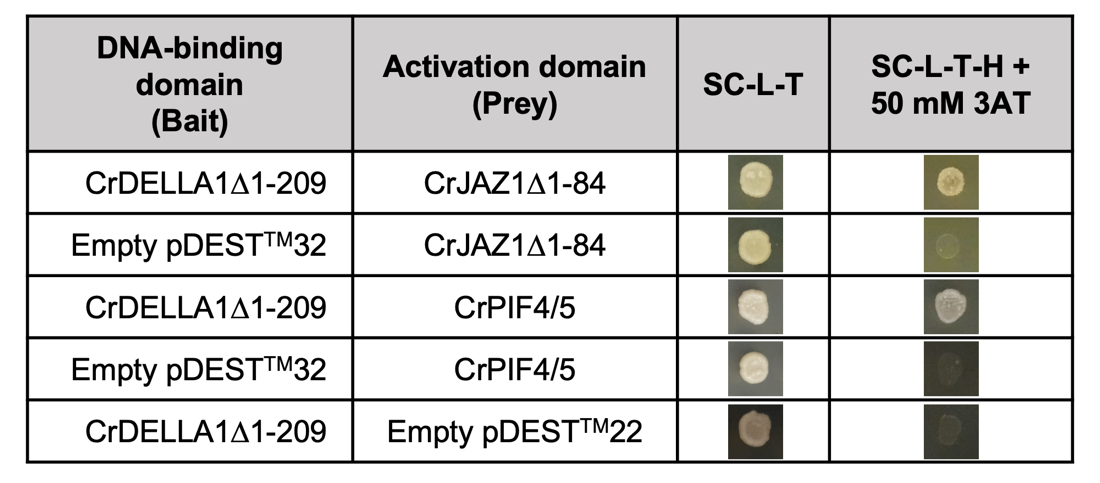
**

**Figure S2.** **CrDELLA1∆1-209 can interact with CrJAZ1 and CrPIF4/5 in a yeast-two hybrid assay.** CrDELLA1 was truncated (amino acids 1-209 removed) to remove self-activation (de Lucas et al., 2008; Hou, Lee, Xia, Yan, & Yu, 2010) and was cloned into the pDEST^TM^32 bait plasmid containing the GAL4 DNA binding domain. CrJAZ1 and CrPIF4/5 were cloned into the pDEST^TM^22 prey plasmid containing the GAL4 activation domain. Growth on synthetic complete media lacking leucine and tryptophan (SC-L-T) serves as a positive control. Growth on synthetic complete media lacking leucine, tryptophan, and histidine with 50 mM 3-aminotriazole (SC-L-T-H + 50 mM 3AT) indicates a positive protein-protein interaction.


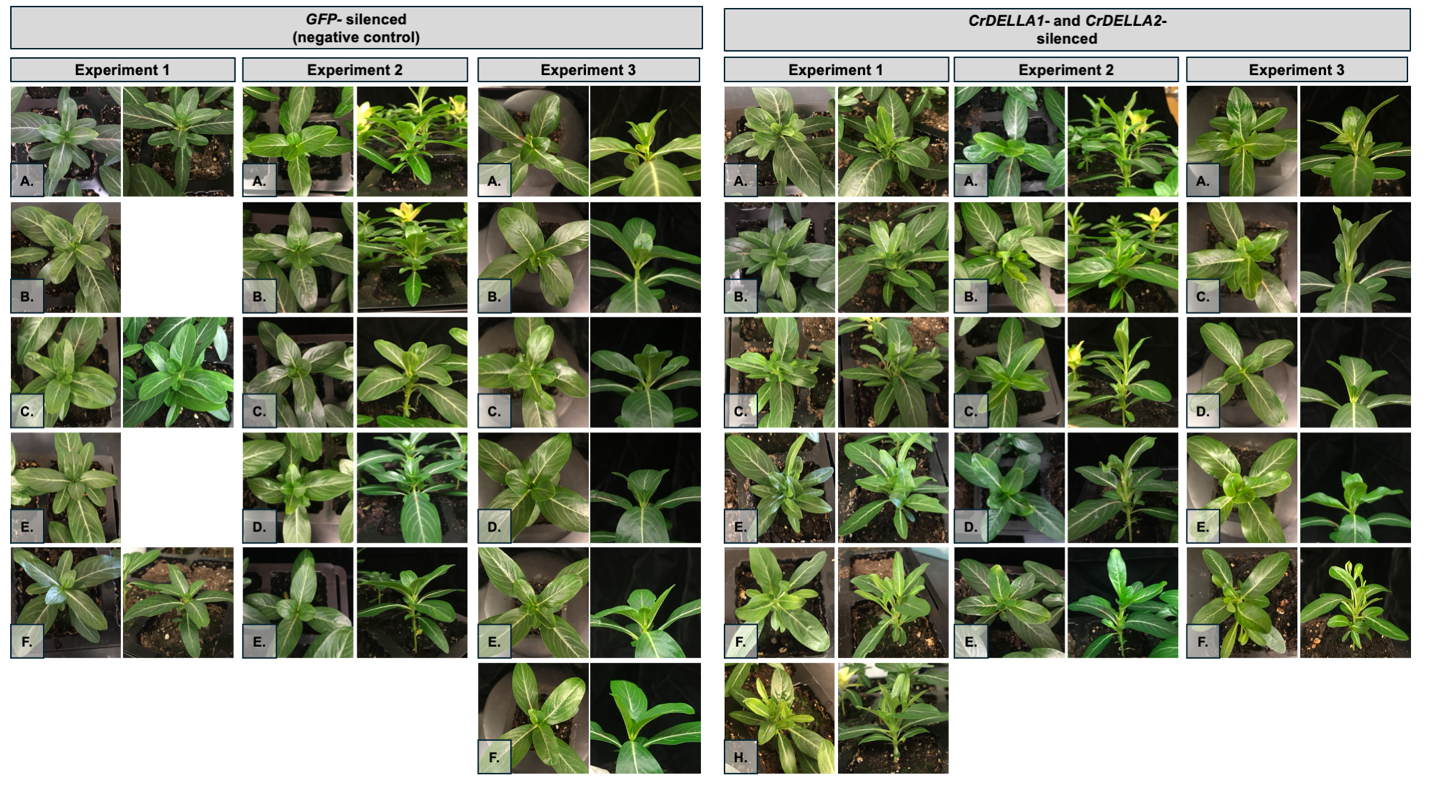


**Figure S3. Collage of *CrDELLA1*- and *CrDELLA2*-silenced plants** and their respective *GFP*-silenced controls for the three experiments analyzed in this study. Top and side view of each plant is shown (A – H; 5 – 6 plants). Plants were confirmed to be *CrDELLA-*silenced using qPCR.


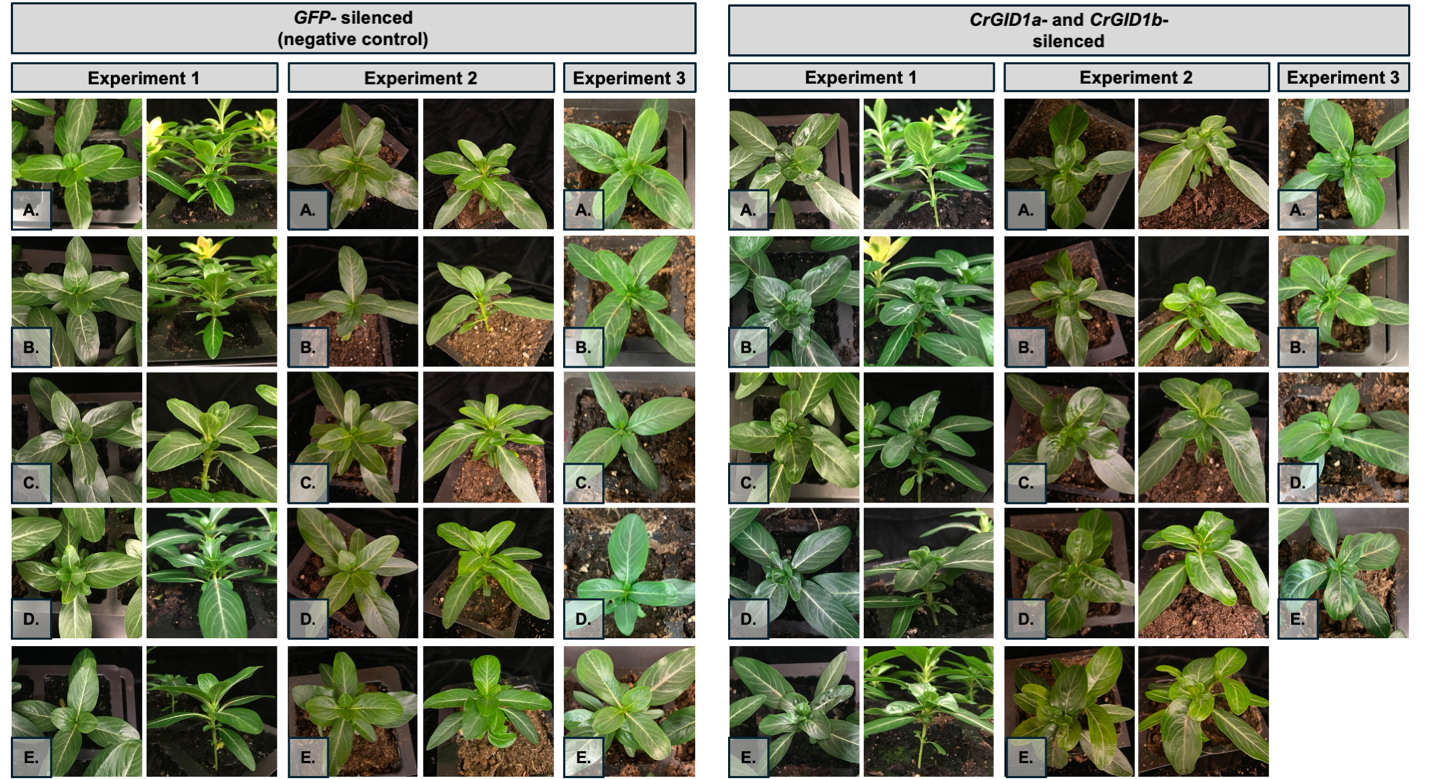


**Figure S4.** **Collage of *CrGID1a*- and *CrGID1b*- silenced plants** and their respective *GFP*- silenced controls for the three experiments analyzed in this study. Top and side view of each plant is shown (A – E; 4 – 5 plants). Plants were confirmed to be *CrGID1-*silenced using qPCR.


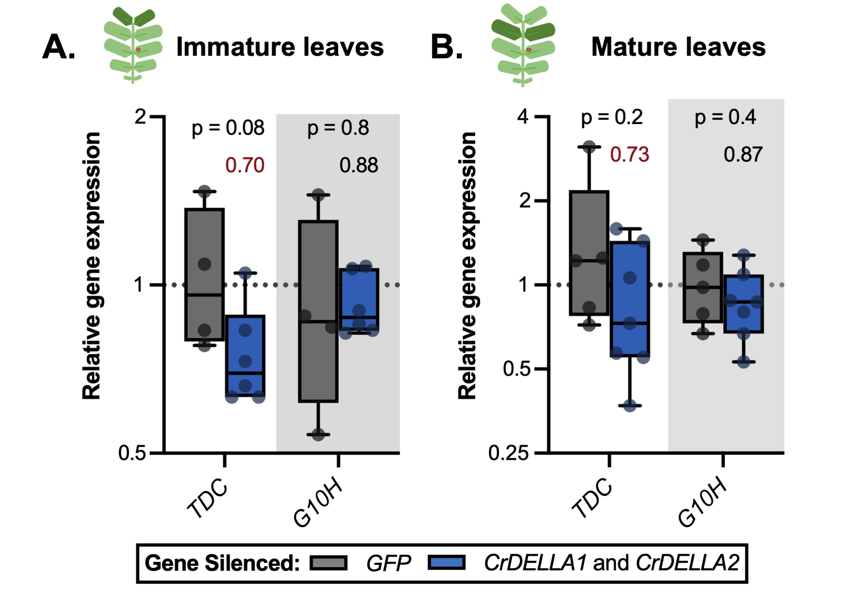


**Figure S5. In the first experiment, silencing *CrDELLA1* and *CrDELLA2* did not significantly impact expression of upstream TIA pathway genes, *TDC* or *G10H* in (A) immature leaves or (B) mature leaves.** RNA was extracted from one immature or one mature leaf for each plant (n = 4-7). Relative gene expression was measured with qPCR and calculated using the 2^-∆∆Ct^ method (Livak & Schmittgen, 2001) relative to the non-targeting negative control condition (*GFP*-silenced plants) and normalized relative to the housekeeping gene, *SAND* (Pollier et al., 2014). Numbers above the boxes represent the median fold change relative to *GFP*-silenced plants (>1.2 is in green, <0.85 is in red). P-values were calculated from an unpaired two-tailed t-test on ∆∆Ct values. Boxes represent the 25^th^ and 75^th^ percentile of each experiment with a line marking the median. Whiskers extend to the minimum and maximum.


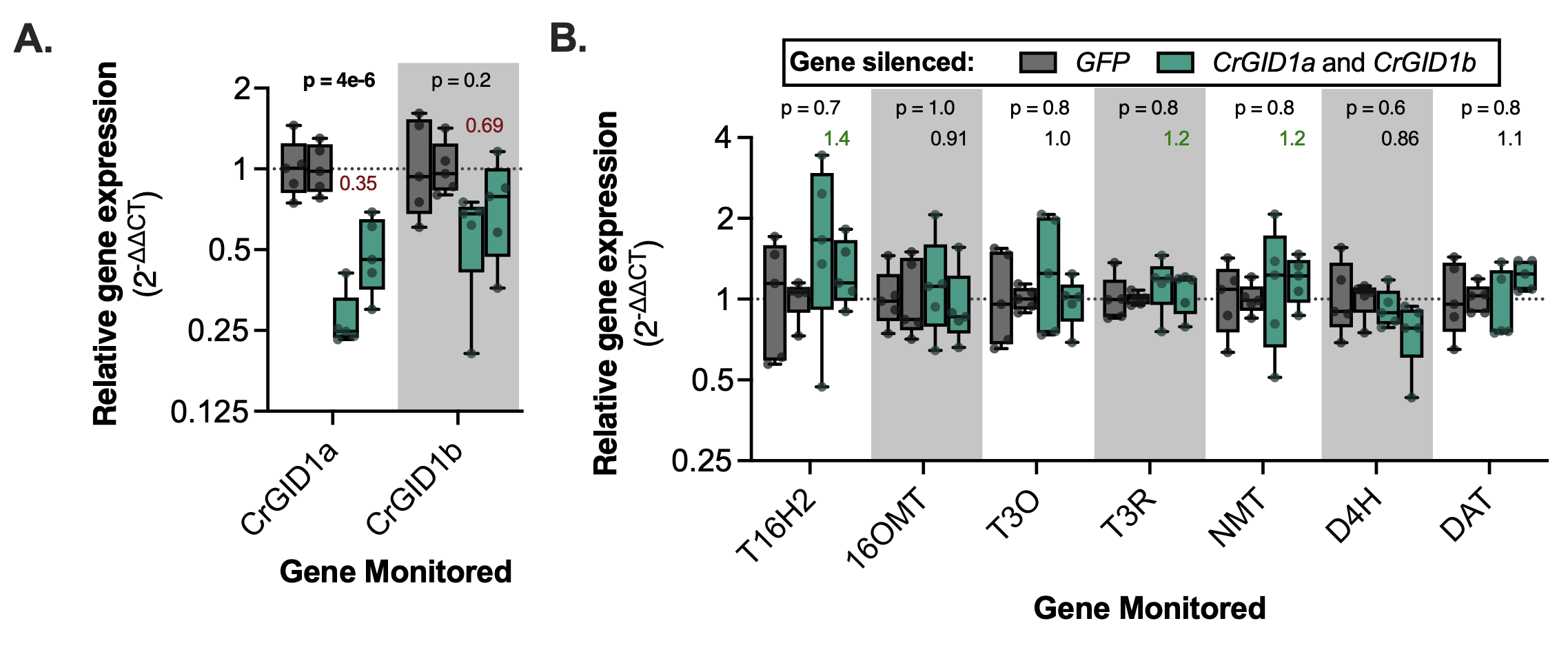


**Figure S6. Silencing *CrGID1a* and *CrGID1b* had no impact on vindoline pathway gene expression in immature leaves.** **(A)** *CrGID1a* but not *CrGID1b* was significantly silenced in *C. roseus* immature leaves. **(B)** Vindoline pathway gene expression was unaffected by *CrGID1a* and *CrGID1b* silencing in immature leaves. RNA was extracted from one immature leaf for each plant (n = 5-7). The experiment was repeated twice (displayed as separate but adjacent boxes). These experiments correspond to the first two experiments analyzed in mature leaves in Figure 6B. Relative gene expression was measured with qPCR and calculated using the 2^-∆∆Ct^ method (Livak & Schmittgen, 2001) relative to the non-targeting negative control condition (*GFP*-silenced plants) and normalized relative to the housekeeping gene, *SAND* (Pollier et al., 2014). Numbers above the boxes represent the median for all of the experiments combined (>1.2 is in green, <0.85 is in red). P-values indicate significance of the effect of gene-silencing according to a two-way ANOVA on ∆∆Ct values, using a full-factorial model for variables “gene-silenced” and “experimental repeat”. P-values were corrected for false discovery rate (FDR = 5%). P-values less than 0.05 are bolded. Complete ANOVA results can be found in Supplemental data 1. Boxes represent the 25^th^ and 75^th^ percentile of each experiment with a line marking the median. Whiskers extend to the minimum and maximum.


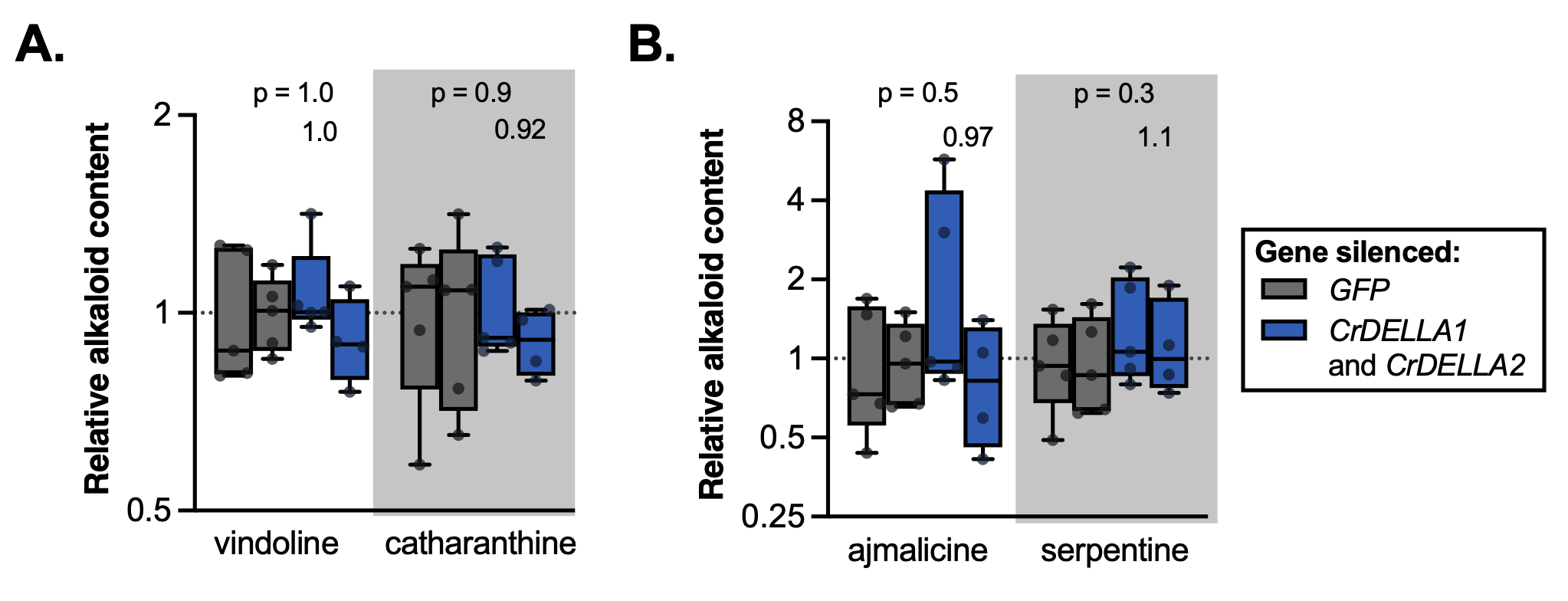


**Figure S7**. **Silencing** ***CrDELLA1* and *CrDELLA2* does not impact terpenoid indole alkaloid (TIA) accumulation in *C. roseus* immature leaves in two experimental repeats. (A)** In immature leaves, vindoline and catharanthine accumulation was unaffected by *CrDELLA*-silencing. **(B)** In immature leaves, ajmalicine and serpentine accumulation was unaffected by *CrDELLA*-silencing. Alkaloids were extracted from one immature or one mature leaf for each plant and analyzed using HPLC-MS-MS. Relative alkaloid contents were calculated from peak areas normalized to an internal standard, and then normalized to the *GFP*-silenced (negative control) condition for each experiment. Each replicate is from an individual plant (n = 4-5). The experiment was repeated twice (displayed as separate but adjacent boxes). These are the same first two silencing experiments analyzed with qPCR (Figures 5 and 6). Numbers above the boxes represent the median fold change relative to *GFP*-silenced plants (>1.2 is in green, <0.85 is in red). P-values indicate significance of the effect of gene-silencing according to a two-way ANOVA on ∆∆Ct values, using a full-factorial model for variables “gene-silenced” and “experimental repeat”. P-values were corrected for false discovery rate (FDR = 5%). P-values less than 0.05 are bolded. Complete ANOVA results can be found in Supplemental data 1. Boxes represent the 25^th^ and 75^th^ percentile of each experiment with a line marking the median. Whiskers extend to the minimum and maximum.


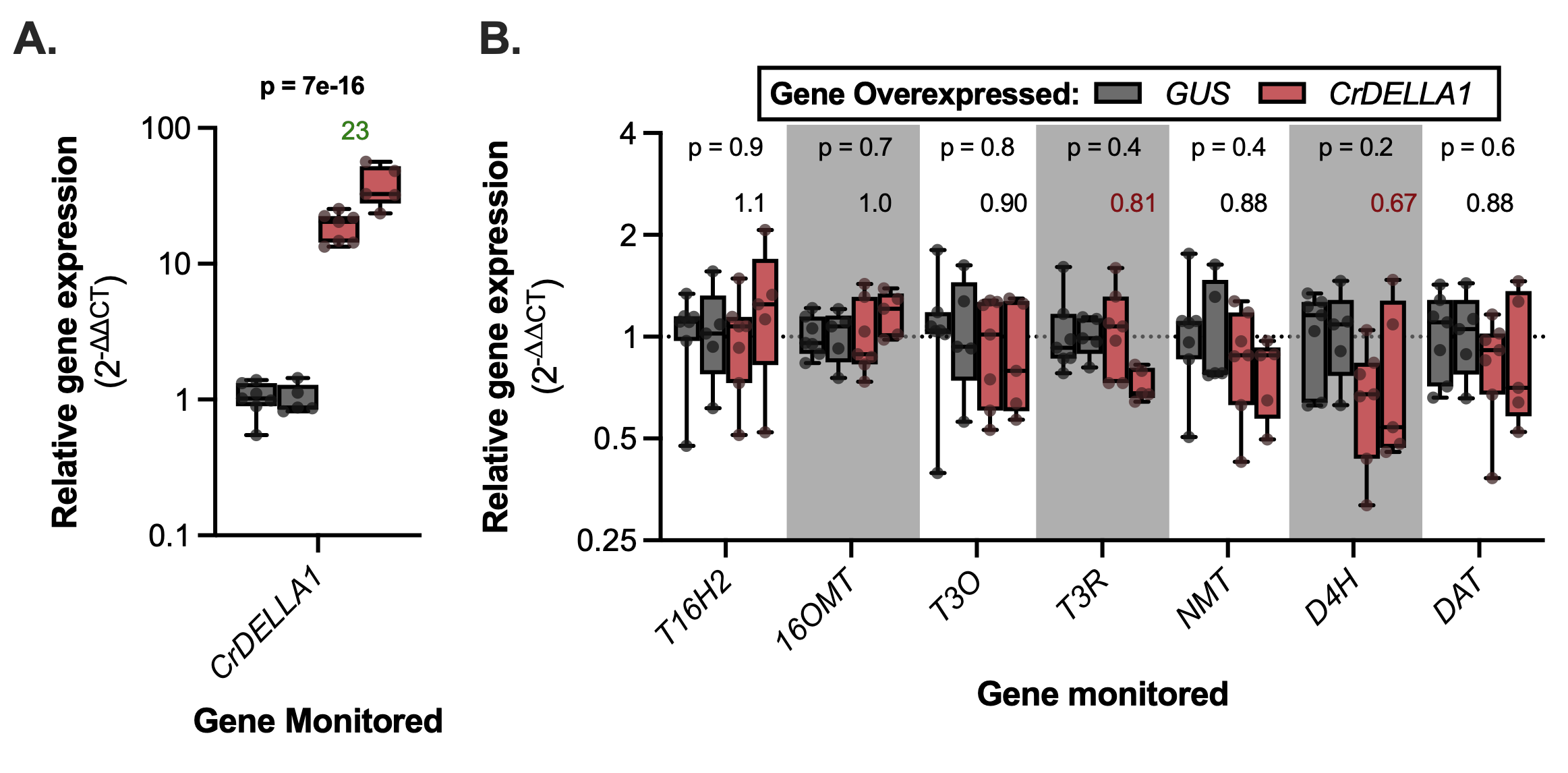


**Figure S8. Overexpression of full-length *CrDELLA1* had no impact on vindoline pathway transcript levels.** Seedlings were transformed with one strain of *A. tumefaciens* (OD_600_=0.2) containing a CaMV2x35s driven *CrDELLA1* or *GUS* (negative control). **(A)** *CrDELLA1* transcript levels increased significantly in the overexpressed samples. **(B)** Vindoline pathway transcripts were unaffected by *CrDELLA1* overexpression. Each biological replicate is a pool of cotyledons isolated from 5 seedlings (n = 5), and the experiment was repeated twice (displayed as separate but adjacent boxes). Relative gene expression was measured with qPCR and calculated using the 2^-∆∆Ct^ method (Livak & Schmittgen, 2001) relative to the control condition (*GUS*-overexpressed seedlings), and normalized relative to the housekeeping gene, *SAND* (Pollier et al., 2014). Numbers above the boxes represent the median for all of the experiments combined (>1.2 is in green, <0.85 is in red). P-values indicate significance of the effect of *CrDELLA1-*overexpression according to a two-way ANOVA on ∆∆Ct values, using a full-factorial model for variables “gene overexpressed” and “experimental repeat”. P-values were corrected for false discovery rate (FDR = 5%). P-values less than 0.05 are bolded. Complete ANOVA results can be found in Supplemental data 1. Boxes represent the 25^th^ and 75^th^ percentile with a line marking the median. Whiskers extend to the minimum and maximum.

**References**

Cole-Osborn, L. F., McCallan, S. A., Prifti, O., Abu, R., Sjoelund, V., & Lee-Parsons, C. W. T. (2024). The role of the Golden2-like (GLK) transcription factor in regulating terpenoid indole alkaloid biosynthesis in Catharanthus roseus. *Plant Cell Reports*, *43*(6), 141. https://doi.org/10.1007/s00299-024-03208-9

Cole-Osborn, L., MCCallan, S., Prifti, O., Abu, R., Sjoelund, V., & Lee-Parsons, C. (2022). KBase Narrative - *C. roseus* Tissue Specificity Analysis using Góngora-Castillo Transcripts and Franke v2 Genome. United States. https://doi.org/10.25982/95510.53/2310383

de Lucas, M., Davière, J.-M., Rodríguez-Falcón, M., Pontin, M., Iglesias-Pedraz, J. M., Lorrain, S., … Prat, S. (2008). A molecular framework for light and gibberellin control of cell elongation. *Nature*, *451*(7177), 480–484. https://doi.org/10.1038/nature06520

Franke, J., Kim, J., Hamilton, J. P., Zhao, D., Pham, G. M., Wiegert-Rininger, K., … O’Connor, S. E. (2019). Gene discovery in Gelsemium highlights conserved gene clusters in monoterpene indole alkaloid biosynthesis. *ChemBioChem*, *20*(1), 83–87. https://doi.org/10.1002/cbic.201800592

Góngora-Castillo, E., Childs, K. L., Fedewa, G., Hamilton, J. P., Liscombe, D. K., Magallanes-Lundback, M., … Buell, C. R. (2012). Development of Transcriptomic Resources for Interrogating the Biosynthesis of Monoterpene Indole Alkaloids in Medicinal Plant Species. *PLoS ONE*, *7*(12). https://doi.org/10.1371/journal.pone.0052506

Hou, X., Lee, L. Y. C., Xia, K., Yan, Y., & Yu, H. (2010). DELLAs Modulate Jasmonate Signaling via Competitive Binding to JAZs. *Developmental Cell*, *19*(6), 884–894. https://doi.org/10.1016/j.devcel.2010.10.024

Liscombe, D. K., & O’Connor, S. E. (2011). A virus-induced gene silencing approach to understanding alkaloid metabolism in *Catharanthus roseus*. *Phytochemistry*, *72*(16), 1969–1977. https://doi.org/10.1016/j.phytochem.2011.07.001

Livak, K. J., & Schmittgen, T. D. (2001). Analysis of relative gene expression data using real-time quantitative PCR and the 2-ΔΔCT method. *Methods*, *25*(4), 402–408. https://doi.org/10.1006/meth.2001.1262

Pollier, J., Vanden Bossche, R., Rischer, H., & Goossens, A. (2014). Selection and validation of reference genes for transcript normalization in gene expression studies in Catharanthus roseus. *Plant Physiology and Biochemistry*, *83*, 20–25. https://doi.org/10.1016/j.plaphy.2014.07.004
